# Corn seed dataset based on hyperspectral and RGB images

**DOI:** 10.1016/j.dib.2026.112455

**Published:** 2026-01-08

**Authors:** Chao LI, Chen Zhang, Wenbo Zhang, Chengzhen LV, Yaqiang Li, Yufen Wang

**Affiliations:** a School of Information Engineering, Xinxiang Institute of Engineering, Xinxiang, Henan Province 453003, China; b School of Computer Science and Technology, Henan Institute of Science and Technology, Xinxiang, Henan Province 453003, China

**Keywords:** Corn seeds, Hyperspectral imaging, RGB imaging, Variety identification

## Abstract

This study employed an HY-6010-S hyperspectral imaging system, covering a spectral range of 400–1000 nm, combined with an RGB industrial camera to acquire multimodal data. The dataset simulates phenotypic analysis scenarios of maize seeds under controlled laboratory conditions, with the ambient temperature maintained at 20–25°C. Comprehensive testing was conducted using 12 different maize varieties. Approximately 200 seed samples were collected per variety, resulting in a total sample size of about 2400, each subjected to hyperspectral and RGB image acquisition. Preprocessing steps included noise reduction, background removal, band selection, and modality alignment. To ensure the accuracy and reliability of the experimental data, HHIT software and Python were utilized for data processing. This dataset plays a significant role in seed variety classification, phenotypic analysis, precision agriculture, and machine learning applications.

Specifications Table

**Table:** 

Subject	Computer Sciences
Specific subject area	Agricultural hyperspectral imaging.
Type of data	Image.
Data collection	Using an HY-6010-S hyperspectral imaging system under stable illumination from two halogen lamps—positioned at 45-degree angles to ensure uniform lighting and prevent shadows-hyperspectral images of the embryo side of maize seeds from 12 varieties were acquired, along with white reference (using a standard polytetrafluoroethylene white board with 50% reflectivity) and dark reference (captured with the lens cap on) images. The spectral range covered 400–1000 nm. Simultaneously, RGB images were captured using an industrial camera to support subsequent analysis and processing.
Data source location	Organization:Xinxiang Engineering University City: Xinxiang, Henan Country: China
Data accessibility	Repository name: Mendeley Data Data identification number: doi: 10.17632/4n4xbnx8sr.1 Direct URL to data: https://data.mendeley.com/datasets/4n4xbnx8sr/1
Related research article	None

## 1 Value of the Data

• This dataset provides multimodal image data acquired under controlled laboratory conditions for the field of maize seed research, comprising hyperspectral and RGB images of approximately 2400 samples across 12 varieties. These data are of critical value for seed science, precision agriculture, and machine learning applications.?
• It reveals the spectral characteristics associated with the internal chemical composition of seeds through hyperspectral imaging, providing a data foundation for the non-destructive assessment of seed biochemical attributes such as starch content. Combined with the external morphological and textural features presented by the RGB images, this multimodal resource enables researchers to establish correlations between seed phenotype and intrinsic quality, offering scientific support for optimized breeding and variety selection.
• The dataset serves as high-quality benchmark data for the automatic identification and purity verification of seed varieties. Researchers can utilize this dataset to develop and validate advanced deep learning models for rapid and accurate variety classification, which is crucial for seed quality control and intellectual property protection.
• In the context of seed vigor assessment and germination rate prediction, this dataset simulates standardized phenotyping scenarios. The analysis of hyperspectral data allows for the identification of early features related to seed vigor, facilitating the development of predictive models. This can assist farmers in selecting high-vigor seeds, thereby improving field emergence rates and crop yield.
• Furthermore, this dataset can function as a valuable resource for scientific research and education, aiding researchers and students in gaining a deeper understanding of the application of multimodal imaging technology in agricultural phenotyping. The analysis and mining of this data can promote interdisciplinary research progress in seed science, computer vision, and smart agriculture.
• Ultimately, the public release of this dataset will contribute to the promotion of sustainable agricultural development. By supporting the selection of superior varieties and the production of high-quality seeds, this resource helps enhance crop production efficiency and resilience from the source, contributing to food security.
• Although the dataset focuses on twelve commercial maize varieties predominant in the Huang-Huai-Hai Plain—one of the world's most critical temperate maize production zones—its scientific value extends far beyond regional interests. These varieties were strategically selected to represent a diverse genetic spectrum of specialty maize (sweet and waxy types), which are globally essential commodities for both human consumption and industrial processing. By capturing the high morphological similarity and subtle spectral divergence inherent in these varieties, this dataset serves as a rigorous benchmark for developing robust computer vision algorithms. Such algorithms are urgently needed worldwide to replace destructive and time-consuming chemical assays in seed purity testing and quality control. Furthermore, the multi-modal alignment (HSI and RGB) protocols established in this study provide a standardized framework that can be readily adapted to maize phenotyping programs across different continents, thereby contributing to global food security and the advancement of digital agriculture.

## 2 Background

Maize, as a vital global crop for food, feed, and industrial raw materials, plays a critical role in ensuring global food security and agricultural economic stability. The seed, being the starting point of agricultural production, directly determines crop emergence rate, growth vigor, and ultimately yield, through its purity, viability, and health status. Therefore, achieving efficient and non-destructive detection of seed quality represents a core challenge in the agricultural sector.

In recent years, the rapid evolution of imaging systems and image processing techniques has fundamentally transformed the landscape of agricultural monitoring and material assessment [1]. Unlike traditional destructive testing, modern imaging technologies offer a non-invasive, high-throughput, and objective alternative for evaluating complex biological structures [2]. Specifically, advances in image processing applications have proven highly effective for assessing leafy materials and other plant tissues, enabling the detection of subtle physiological changes that are invisible to the human eye [3]. As we look toward the future of imaging technology, the integration of multi-modal data and sophisticated processing algorithms is expected to further enhance the precision of quality control in the food and seed industries [4]. These technologies not only reduce labor costs but also provide spatially resolved information that is critical for understanding the distribution of internal constituents, such as starch and moisture, within individual kernels. Hyperspectral imaging (HSI) has emerged as a transformative technology in the agricultural and food sectors, offering the unique ability to integrate spatial and spectral information simultaneously [4]. Researchers worldwide have conducted extensive explorations in this field. For instance, in variety classification, the SSATNet model proposed by Wang et al. [5] and the 1D-CNN-LSTM-Attention model employed by Zhang et al. [6] both achieved high accuracy exceeding 95%, demonstrating the significant potential of HSI combined with deep learning models. One of the primary advantages of HSI is its capacity for non-destructive, rapid, and high-throughput analysis, which is essential for real-time quality monitoring [4,6]. Recent studies have demonstrated the versatility of HSI in detecting complex chemical compositions and physical properties across various food matrices. For instance, HSI combined with artificial neural networks has been successfully applied to detect specific elements like chlorine in chemical fertilizers [7].In the field of nut quality assessment, HSI has proven to be a robust tool for evaluating internal defects and chemical constituents [8].Furthermore, the integration of HSI with advanced machine learning methods has significantly improved the precision of quantitative analysis. Recent research has shown its efficacy in estimating ash content in wheat flour [9] and classifying different wheat flour types based on their unique spectral signatures [10]. Beyond solids, HSI has also demonstrated exceptional performance in predicting complex biochemical parameters in liquid or semi-liquid products, such as predicting sucrose, proline, and the fructose/glucose ratio in date syrup [10]. These diverse applications highlight that HSI, as a powerful sensing modality, not only surpasses traditional RGB imaging in chemical sensitivity but also provides a more comprehensive understanding of agricultural product quality, paving the way for its wider industrial adoption [4].In seed vigor detection, Wang et al. [11] and Liu et al. [12] utilized optimization algorithms and 1D-CNN models, respectively, for vigor identification, achieving accuracies of up to 97.23%. Furthermore, HSI has also showcased remarkable performance in non-destructive biochemical indicator detection [[13], [14]] and disease detection [15]. Despite these fruitful research outcomes, a review of existing literature reveals critical bottlenecks hindering further development in the field. Firstly, limitations exist in the diversity and availability of datasets; many studies rely on small-scale or lab-specific data, lacking broad representativeness. Secondly, the vast majority of research depends on single-modal HSI data, lacking systematic integration with RGB imaging which provides rich morphological and textural information. This limits the comprehensive utilization of multi-dimensional features. Finally, the absence of standardized data acquisition and preprocessing protocols challenges the model generalizability and comparability of results across different studies.

Consequently, there is a pressing need for a large-scale, standardized benchmark dataset incorporating multimodal image resources to support the development and validation of next-generation agricultural AI models. To address this gap, we present this dataset. By providing paired hyperspectral and RGB images from 12 maize varieties, acquired and preprocessed under strictly controlled conditions, this dataset aims to serve as a robust resource for researchers to explore key features related to seed purity, vigor, and phenotype, ultimately fostering innovation in precision breeding, seed quality control, and sustainable agriculture.

## 3 Data Description

**Fig. 1:**
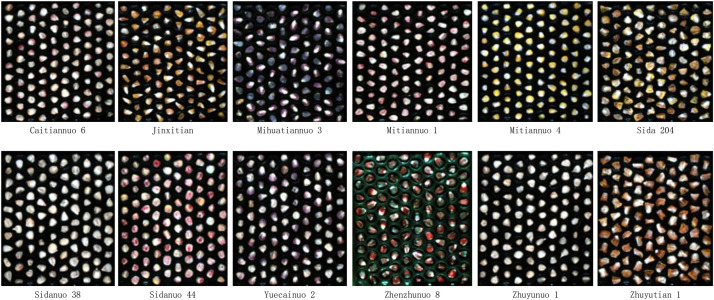
Sample of the data.

The maize seed samples were selected from the following predominant commercial varieties: Caitiannuo 6, Jinxi Tiantian, Mihua Tiannuo 3, Mitiannuo 1, Mitiannuo 4, Sida 204, Sidanuo, Sidanuo 38, Yuecainuo 2, Zhenzhunuo 8, Zhuyunuo 1, and Zhuyutian 1, as detailed in Fig. 1. These varieties primarily represent sweet corn and waxy corn types, sourced from multiple regions in China including Henan and Guangdong, ensuring comprehensive representation of phenotypic diversity—encompassing variations in morphology, color, and texture—with detailed characteristics provided.

The maize seed samples comprise twelve predominant commercial varieties, each with distinct agronomic characteristics, the relevant information is shown in Table 1.

**Table 1:** Variety description.

Variety	Color	Type	Average yield (kg/acre)	Production area
Caitiannuo 6	Purple and white	Glutinous	867.8	Southeast region (including central and southern Jiangsu), Southwest region
Jinxitian	Yellow	Sweet	1050-1082	Guangdong Province
Mihua Tiannuo 3	Suit	Glutinous and sweet	866-907	North China (Donghua North, Huang Huai Hai)
Mitiannuo 1	White	Glutinous and sweet	1001	Southwest (Sichuan, Guizhou, etc.)
Mitiannuo 4	Suit	Glutinous and sweet	848-908	Donghua North
Sida 204	Yellow	Glutinous	900	North
Sidanuo	Suit	Glutinous	949	North (Donghua North)
Sidanuo 38	Suit	Glutinous	817-900	North China (Donghua North, Huang Huai Hai)
Yuecainuo 2	Purple and white	Glutinous	871	Guangdong, Guangxi, and Southeast regions
Zhenzhunuo 8	Purple	Glutinous	871	Southeast (south of the Huai River in Anhui Province, etc.)
Zhuyunuo 1	White	Glutinous	848-896	Southeast and Southwest
Zhuyutian 1	Yellow and White	Sweet	1035	Guangdong Province

**Caitiannuo 6** requires 80–84 days from emergence to fresh-ear harvest, exhibits a plant height of 210 cm and an ear length of 19 cm, features purple-white bicolor kernels, yields an average of 867.8 kg/mu, and is suitable for Southeastern and Southwestern China.

**Jinxi Tian** has a growth period of 78–85 days in spring and 73–78 days in autumn, reaches 205–207 cm in plant height with ears of 19–19.8 cm, produces yellow kernels, achieves a yield of 1050–1082 kg/mu, and is adapted to Guangdong Province.

**Mihua Tiannuo 3** matures in 88 days in Northeastern China and 72 days in the Huang-Huai-Hai Region, grows to 234 cm/217 cm in different regions with ear lengths of 18.5 cm/17.7 cm, displays variegated kernels, yields 907/866 kg/mu, and is cultivated in Northern China.

**Mitiannuo 1** requires 86.5 days to harvest, stands 217 cm tall with 17.8 cm ears, has white kernels, produces 1001 kg/mu, and is suited to Southwestern China.

**Mitiannuo 4** matures in 85 days in Northeastern China and 74 days in the Huang-Huai-Hai Region, reaches 243 cm/223 cm in height with 19.7 cm/18.8 cm ears, shows variegated kernels, yields 908/848 kg/mu, and is grown across multiple regions.

**Sida 204** needs 79 days to harvest, grows to 218 cm with 21.2 cm ears, has unspecified kernel color, yields 900 kg/mu, and is adapted to Northern China.

**Sidanuo 38** requires 93.6 days, reaches 254 cm in height with 18.9 cm ears, features variegated kernels, yields 949 kg/mu, and is grown in Northern China.

**Sidanuo 44** matures in 89 days in Northeastern China and 75 days in the Huang-Huai-Hai Region, grows to 261 cm/259 cm with 21.2 cm/19.8 cm ears, displays variegated kernels, yields 900/817 kg/mu, and is cultivated in Northern China.

**Yuecainuo 2** requires 83–90 days to harvest, reaches 175–214 cm in height with 17–18.7 cm ears, features purple-white bicolor kernels, yields 871 kg/mu, and is suitable for Southern China.

**Zhenzhunuo 8** needs 79.9 days, grows to 240 cm with 18.1 cm ears, has purple kernels, yields 871 kg/mu, and is adapted to Southeastern China.

**Zhuyunuo 1** matures in 82 days in Southeastern China and 86 days in Southwestern China, reaches 220 cm/230 cm in height with 19.7 cm/19.8 cm ears, features white kernels, yields 896/848 kg/mu, and is grown in Southeastern and Southwestern China.

**Zhuyutian 1** requires 71–72 days in autumn, reaches 196–207 cm in height with 18.6–19.3 cm ears, displays yellow-white bicolor kernels, yields 1035 kg/mu, and is mainly cultivated in Guangdong Province.

**Fig. 2:**
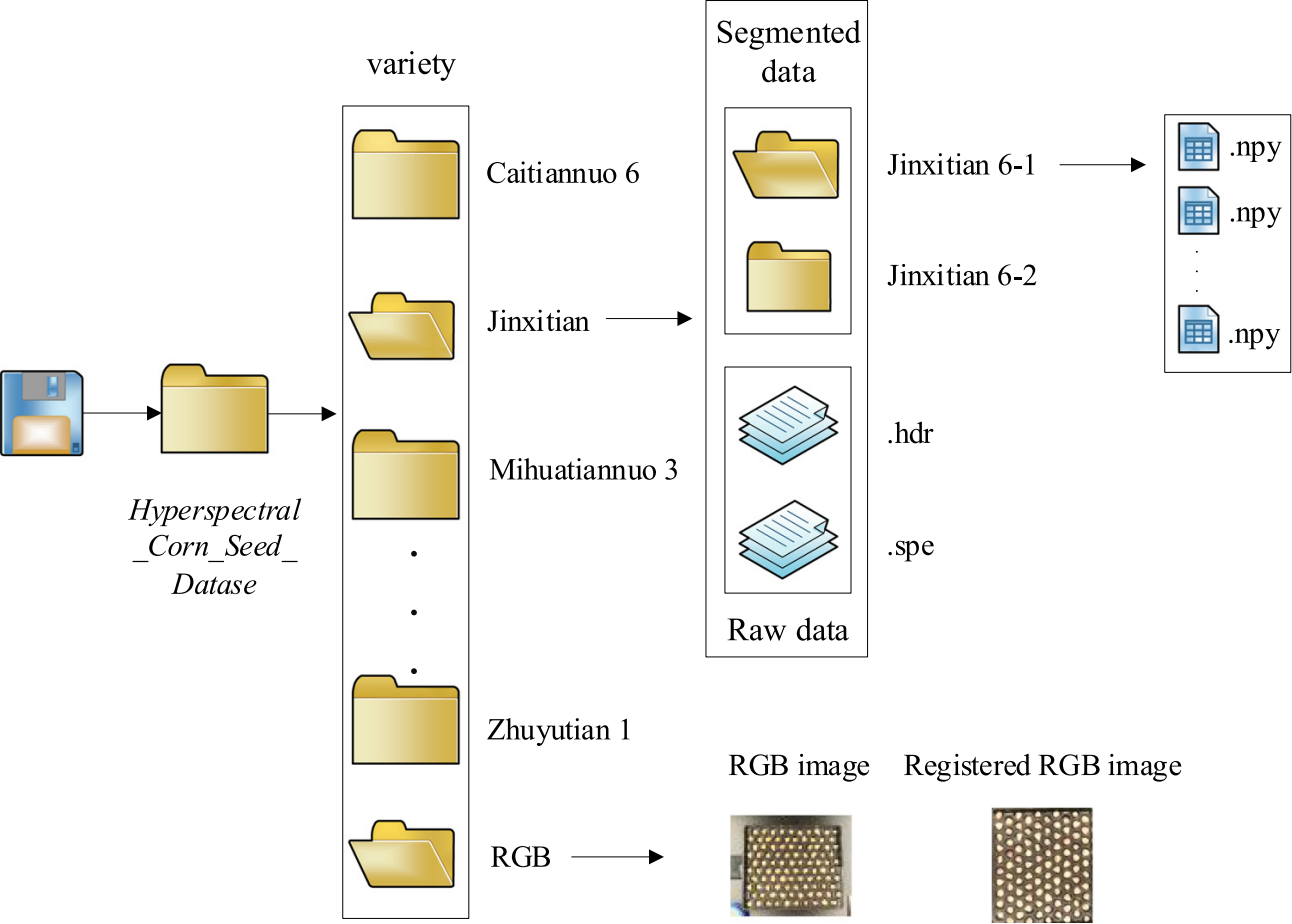
Dataset structure.

To construct the dataset, researchers implemented a rigorous seed selection process, ultimately choosing approximately 200 intact, undamaged, highly viable, and healthy seeds per variety, resulting in a total sample size of 2400 seeds. Post-collection, all seeds were immediately sealed in paper bags to prevent moisture absorption or contamination.The experimental design was tailored for non-destructive data acquisition using both hyperspectral and RGB imaging, supporting subsequent variety classification and phenotypic analysis. All data were collected under controlled laboratory conditions, with ambient temperature maintained between 20-25°C. The push-broom scanning mode was consistently applied throughout image acquisition to ensure data uniformity and reproducibility.The overall directory structure includes parent folders named 'Hyperspectral_Corn_Seed_Datase'. Subfolders are named according to the varieties, each containing original hyperspectral data files (.hdr and .spe) and RGB images (.jpg), The specific structure is shown in Fig. 2. Files are organized by variety for ease of access, comprising a total of 2400 samples. The specific structure is illustrated in the accompanying figure, The specific models of hyperspectral cameras are shown in Table 2.

**Table 2:** Hyperspectral camera information.

Name	Model	Manufacturer	Place of origin	Spectral resolution	Spectral range	Application fields
Portable hyperspectral imaging device	HY-6010-S	Hangzhou Gaopu Imaging Technology Co., Ltd	Hangzhou China	Better than 2.8 nmnm	400-1000nm	Agriculture and forestry research, ecological environment monitoring, urban applications, industrial applications, geological exploration

## 4 Experimental Design, Materials and Methods

The deployment of deep learning and machine learning models critically depends on dataset quality. Specifically, image samples require accurate labeling, balanced class distribution, and comprehensive detail. In this study, the entire pipeline—from data acquisition to segmentation—was systematically planned and executed.

The research initially focused on processed maize kernels, examining their production scale, market popularity, and health implications before investigating their availability, leading sales, and consumption rates in the marketplace. Subsequently, the team surveyed local vendors and suppliers distributing these maize varieties, finalizing the process by documenting local names and verifying corresponding scientific classifications.

Imaging was performed inside a dark chamber to eliminate ambient light interference. Hyperspectral data were acquired using an HY-6010-S visible/near-infrared portable hyperspectral imaging system operating in a push-broom scanning mode. The system covers a spectral range of 400–1000 nm, with a spectral resolution better than 2.8 nm, a sampling interval of 0.5 nm, a native detector resolution of 1920 × 1200 pixels, and an effective bit depth of 12 bits. Simultaneously, RGB images were captured using an industrial camera with a resolution of 4624 × 3472 pixels, enabling multimodal synchronization.

Auxiliary equipment included:

• Twohalogen lamps positioned at 45-degree angles to ensure uniform illumination and avoid shadows, with specific details illustrated in Figure 2.
• A standard polytetrafluoroethylene white board with 50% reflectivity for white reference calibration.
• A lens cap for dark reference acquisition.
• A translation stage for precise sample positioning.
• HHIT software for image acquisition.
• Python for subsequent data processing.

The hardware environment consisted of an Intel Ultra 9 CPU and an NVIDIA RTX 4090D GPU.

**4.1. Data Acquisition Procedure**

**Table:** 

Collection steps	images
***Sample Preparation*** Seed surfaces were cleaned to remove dust or impurities, ensuring no physical damage. For each variety, seeds were oriented with the embryo side facing upward and arranged on a black background or conveyor belt in a 10 × 10 grid layout (100 seeds per batch) to facilitate batch scanning.	
***Instrument Warm-up and Calibration*** The hyperspectral imager and RGB camera were powered on and allowed to warm up for 30 minutes to stabilize performance. A reference panel was positioned for calibration. Key parameters were set as follows: hyperspectral exposure time ≈ 5000 ms, gain = 2, frame rate = 18 Hz, and translation speed = 0.006-0.03 cm/s. The RGB camera was synchronized to ensure spatial alignment.	
***Image Acquisition*** Aligned seed batches were placed on a motorized translation stage, and push-broom (line-scanning) mode was initiated. The hyperspectral imager captured data line-by-line, which was subsequently assembled into a complete hyperspectral cube (≈720 × 480 pixels spatially, 300 spectral bands). Simultaneously, the RGB camera acquired corresponding color images. Each batch was scanned once without disturbing seed positions. This process was repeated for all samples, yielding approximately 2400 hyperspectral cubes and paired RGB images.	
***Spectral Correction*** Relative reflectance was calculated using a standard white reference panel (50% reflectivity) and a dark reference to correct raw images. The correction formula is given by: $I_{c}=\frac{I_{\text{raw}}-I_{\text{dark}}}{I_{\text{white}}-I_{\text{dark}}}\times R_{\text{white}}$ Where $I_{\text{raw}}$ is the raw image,$I_{\text{white}}$ the white reference image, $I_{\text{dark}}$ the dark reference, and$\;R_{\text{white}}$the known reflectance of the reference white. This step minimizes effects of uneven illumination, sensor noise, and dark current, while avoiding potential saturation issues from high-reflectance standards, thereby improving data fidelity for medium-reflectance maize seeds.	
***Seed Segmentation and Spectral ExtractionIn*** ENVI software, a single-band grayscale image (784.8 nm) was extracted from each hyperspectral cube. An Otsu thresholding method was applied to generate a binary mask, enabling seed contour detection and segmentation of individual seed regions of interest (ROIs).	
***Multimodal Data Alignment*** To achieve pixel-level fusion, SIFT (Scale-Invariant Feature Transform) was employed to extract robust keypoints from both the RGB image and a pseudo-color image (synthesized from HSI bands 116, 65, and 37). 333A brute-force matcher combined with Lowe's ratio test (0.75) and the RANSAC algorithm was used to filter out outliers and estimate the homography matrix (M). 4By applying the inverse matrix ($M^{-1}$) and scale factor (S), the high-resolution RGB image was warped and projected onto the HSI spatial coordinate system, ensuring precise multimodal alignment for downstream phenotypic analysis.	
***Data Storage*** Raw hyperspectral data—including white and dark reference sets—were saved in .hdr and .spe formats. RGB images were stored as .jpg files. Accompanying metadata included seed IDs and variety labels.	

## Limitations

The dataset used in this study is primarily composed of region-specific maize varieties, predominantly commercial sweet and waxy maize cultivated in the Huang–Huai–Hai Plain and southern China. As such, it may not comprehensively represent the global genetic and phenotypic diversity of maize seeds, nor fully reflect varieties favored in broader international markets based on consumer preference and commercial popularity.A further limitation lies in the incomplete availability of biochemical and quality-related metadata for certain varieties, owing to the absence of comprehensive and standardized source documentation. Specifically, key chemical attributes that underpin intrinsic seed quality—including protein content, crude fat content, precise moisture content at the time of data acquisition, starch purity, and dietary fiber content—were not systematically recorded. This deficiency constrains the direct quantitative linkage between multimodal imaging features (both spectral and morphological) and the underlying biochemical composition of the seeds, thereby limiting the potential to develop more accurate and mechanistically interpretable predictive models for seed quality assessment.In addition, nutritional information obtained from commercial platforms, such as partial nutrient content descriptions for locally marketed varieties, is often presented without verifiable or authenticated sources. This further hampers the rigorous validation of imaging-based inferences regarding seed quality and nutritional characteristics.Although substantial efforts were made to ensure the reliability, consistency, and relevance of the dataset within the scope of the present study, these limitations underscore the necessity for future research to establish more extensive and systematic datasets. In particular, the coordinated acquisition of paired multimodal imaging data and standardized biochemical measurements across diverse regional and commercial maize varieties will be critical for advancing robust, generalizable, and biologically grounded seed quality evaluation frameworks.

## Ethics Statement

The ethical prerequisites for publishing in Data in Brief have been thoroughly reviewed and adhered to. It is hereby validated that the present study does not entail the participation of animal experimentation, human subjects, or the utilization of any data acquired from social media platforms.

## Credit Author Statement

Chao Li: Methodology, Writing original draft, Investigation. Chen Zhang: Software, Validation, Writing review & editing. Wenbo Zhang: Validation, Writing review & editing. Yaqiang Li: Validation, Writing review & editing. Chengzhen Lü: Validation, Writing review & editing. Yufen Wang: Supervision, Validation, Writing review & editing.

## Data Availability

Mendeley DataHyperspectral_corn_seed_dataset (Original data).

## Appendix Supplementary materials

Image, application 1
