# Supplementary figures and images for: Corn seed dataset based on hyperspectral and RGB images
Source: Data Brief. 2026 Jan 8;65:112455. doi: 10.1016/j.dib.2026.112455 (PMC12856158; doi:10.1016/j.dib.2026.112455)

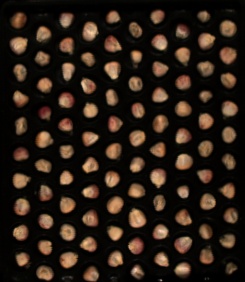

Supplement: Supplementary file 1 [file mmc1.zip › Spatial Registration/Caitiannuo No.6-1/Caitiannuo No.6-1-hsi.jpg]

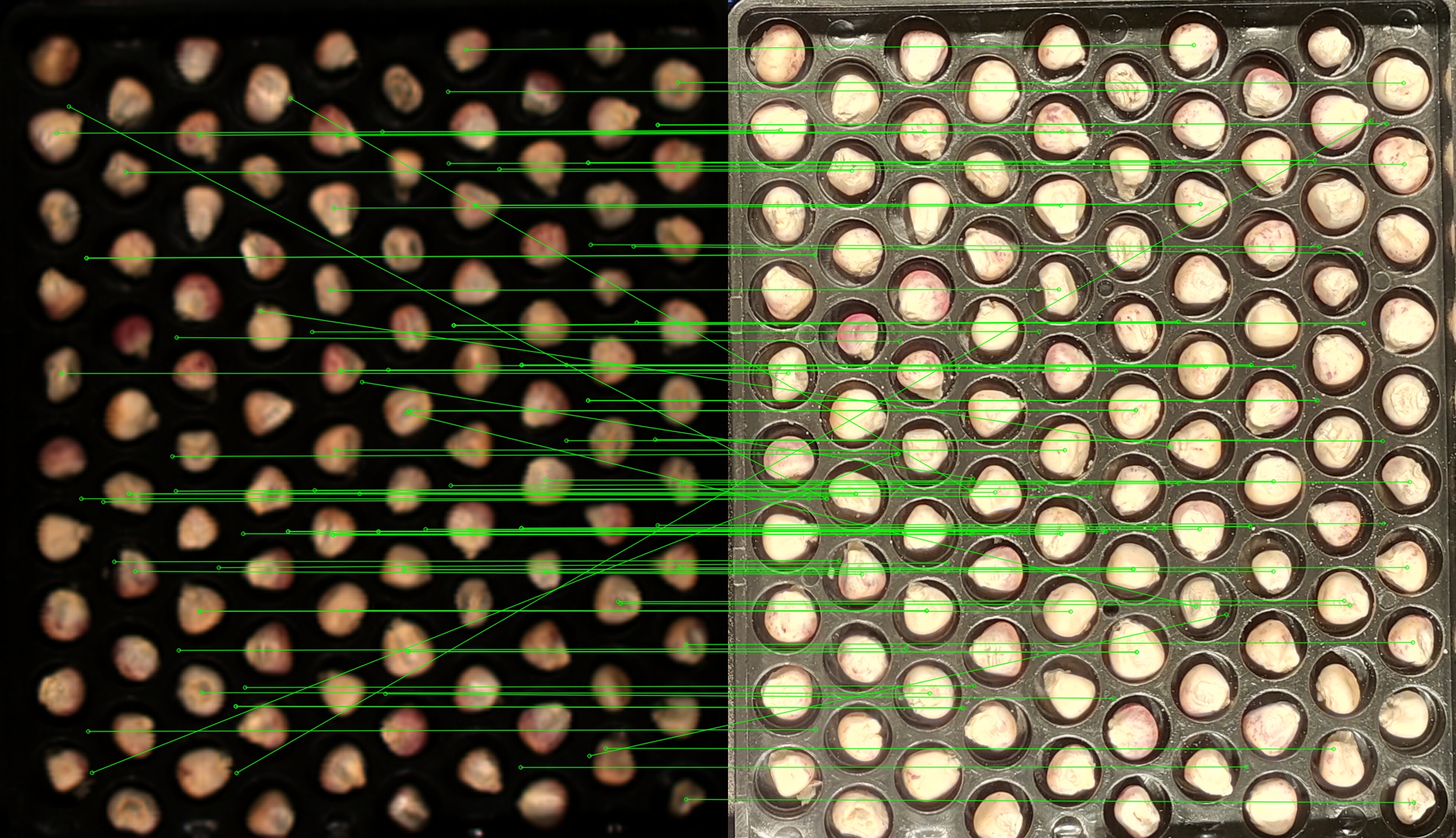

Supplement: Supplementary file 1 [file mmc1.zip › Spatial Registration/Caitiannuo No.6-1/Caitiannuo No.6-1-matches.jpg]

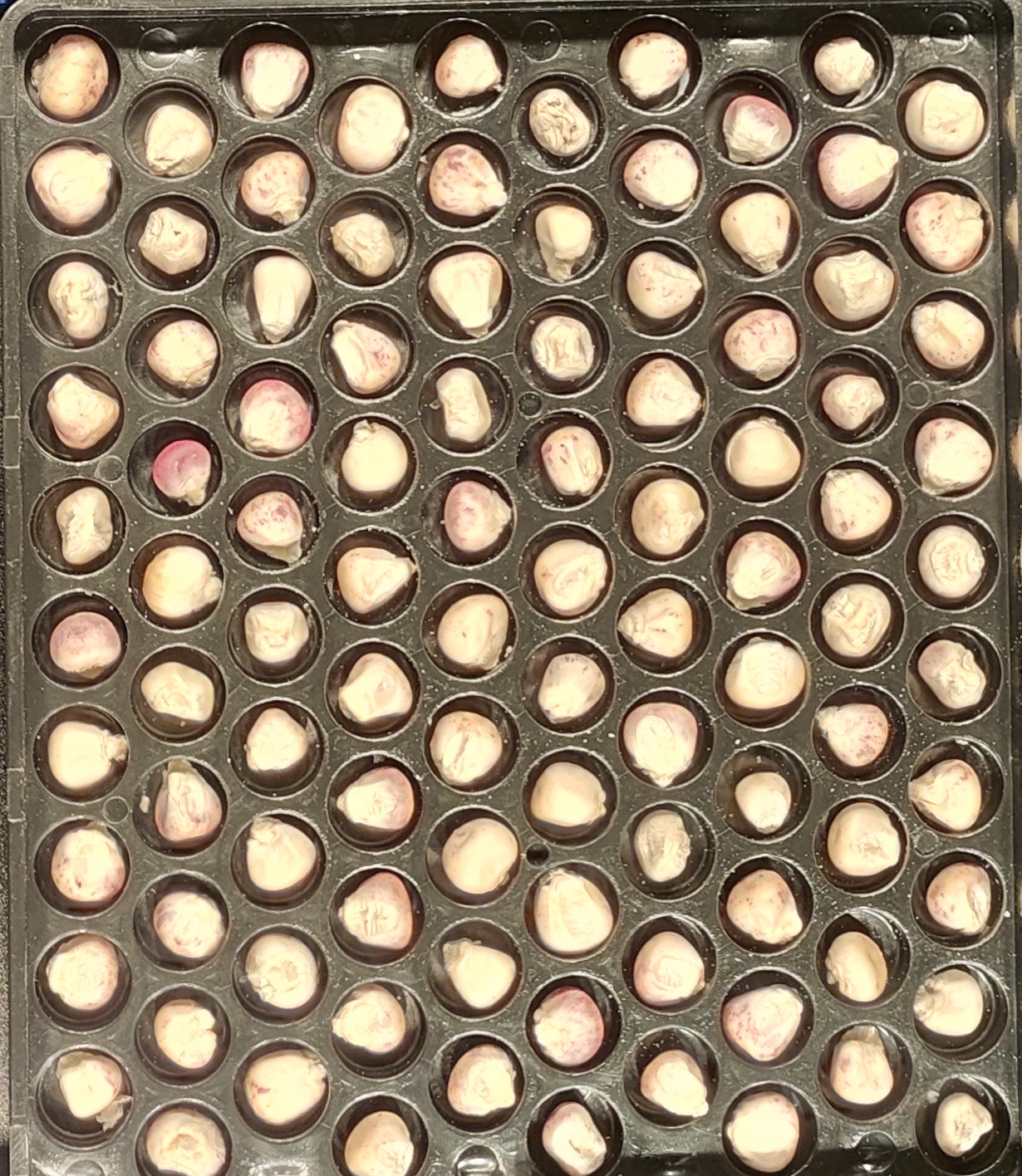

Supplement: Supplementary file 1 [file mmc1.zip › Spatial Registration/Caitiannuo No.6-1/Caitiannuo No.6-1-rgb.jpg]

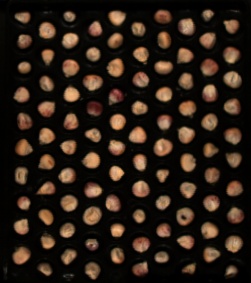

Supplement: Supplementary file 1 [file mmc1.zip › Spatial Registration/Caitiannuo No.6-2/Caitiannuo No.6-2-hsi.jpg]

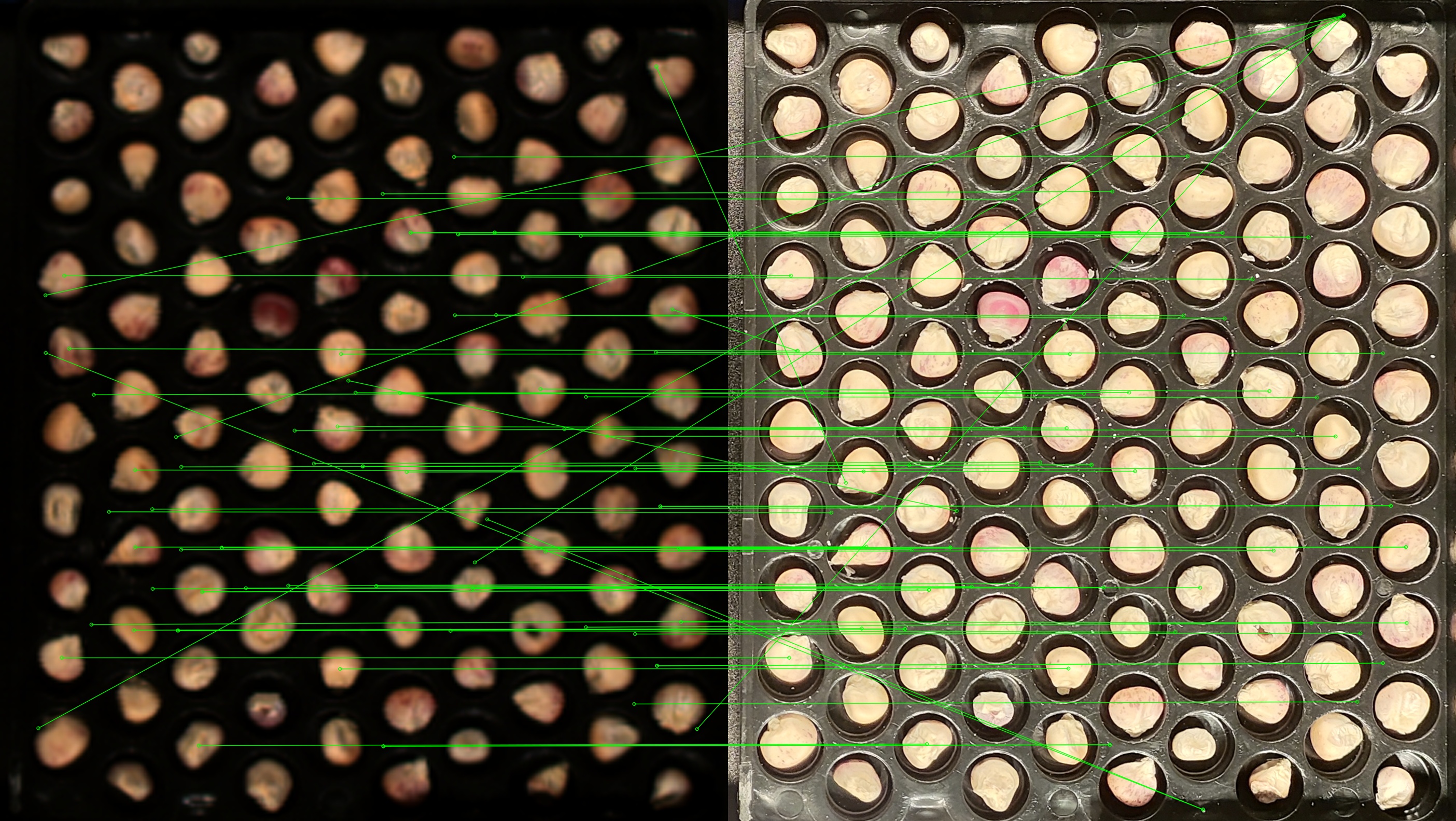

Supplement: Supplementary file 1 [file mmc1.zip › Spatial Registration/Caitiannuo No.6-2/Caitiannuo No.6-2-matches.jpg]

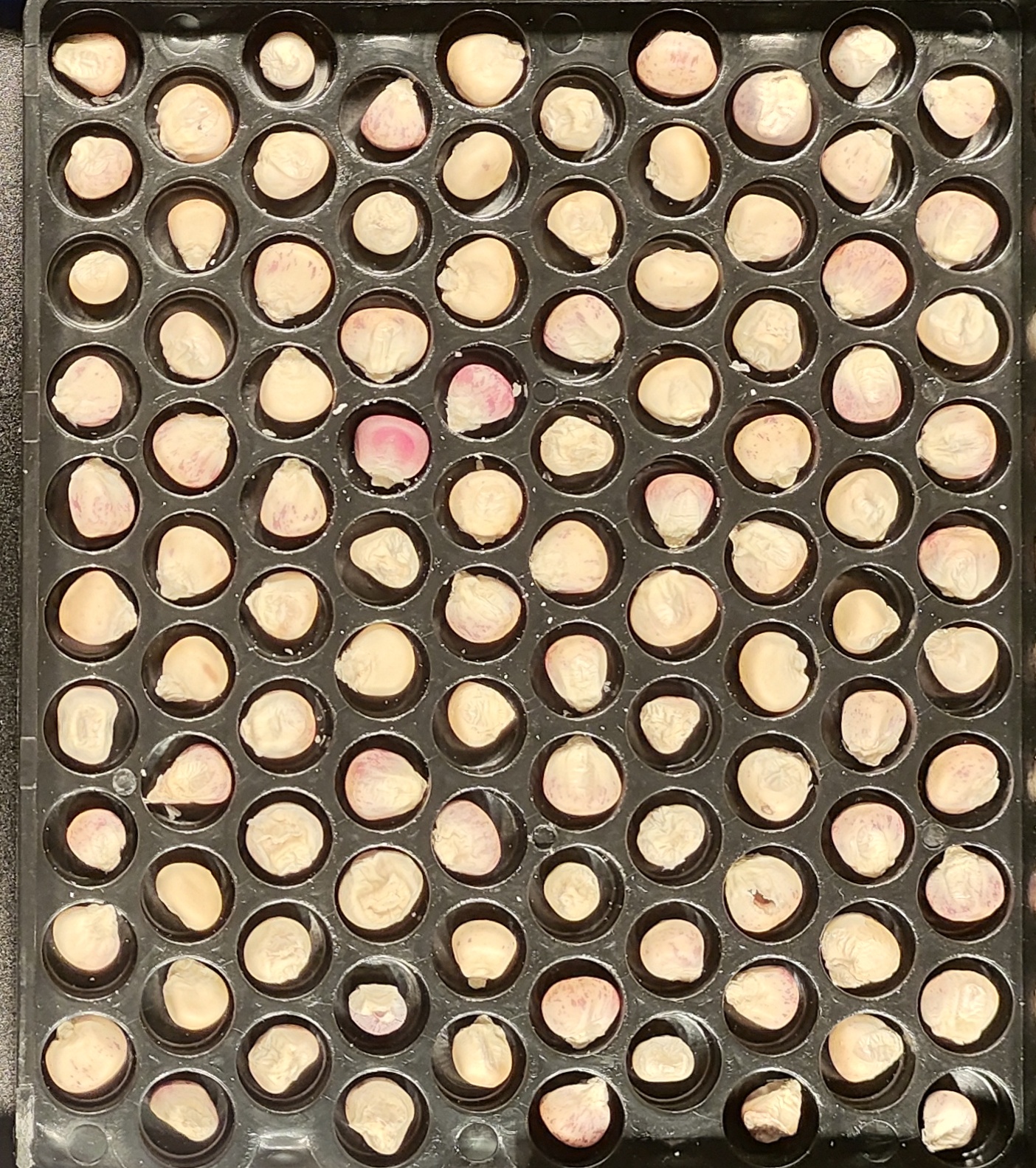

Supplement: Supplementary file 1 [file mmc1.zip › Spatial Registration/Caitiannuo No.6-2/Caitiannuo No.6-2-rgb.jpg]

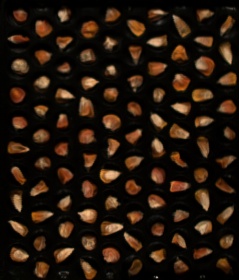

Supplement: Supplementary file 1 [file mmc1.zip › Spatial Registration/Jinxitian-1/Jinxitian_2_hsi_pseudo.jpg]

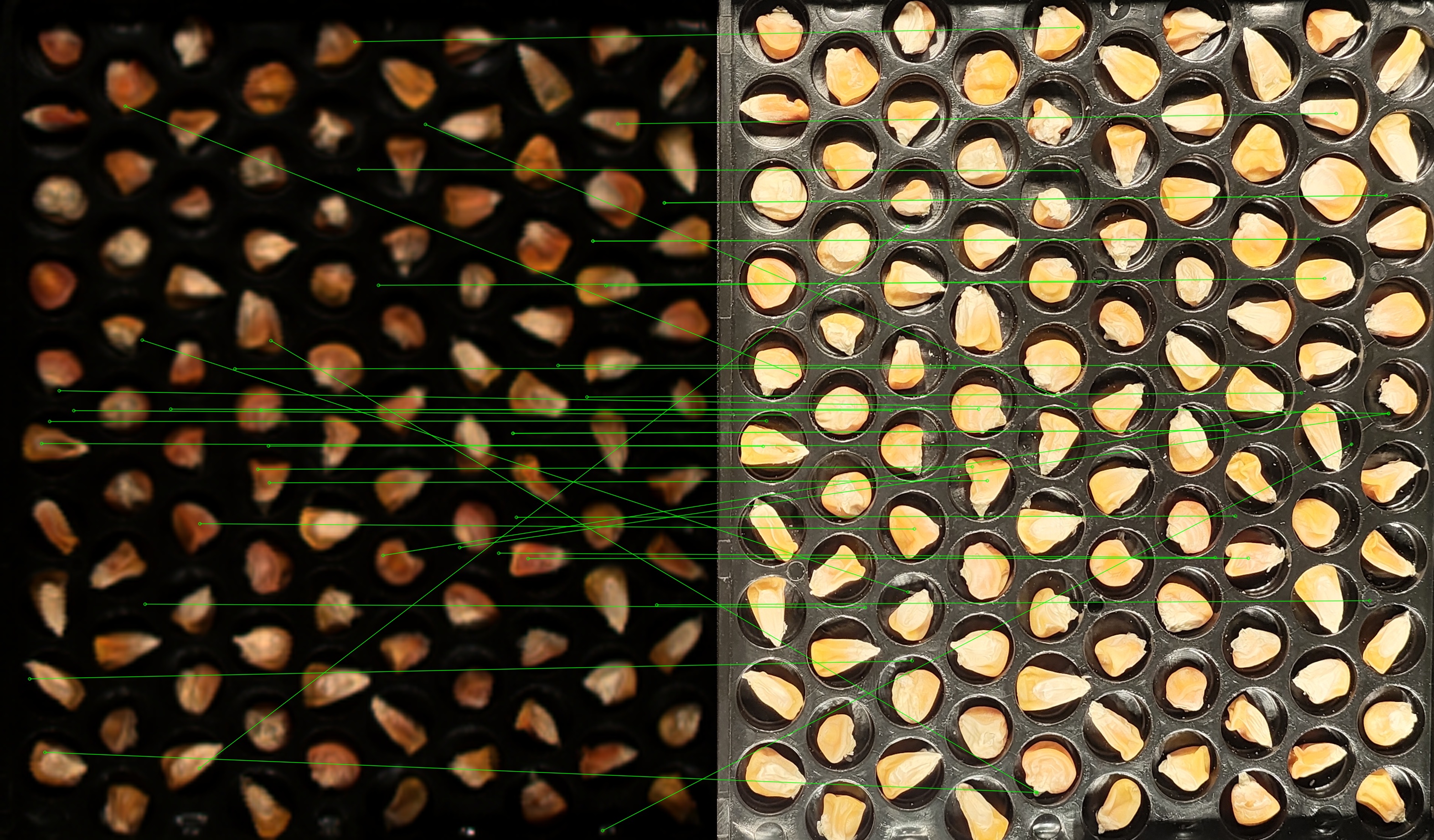

Supplement: Supplementary file 1 [file mmc1.zip › Spatial Registration/Jinxitian-1/Jinxitian_2_matches_viz.jpg]

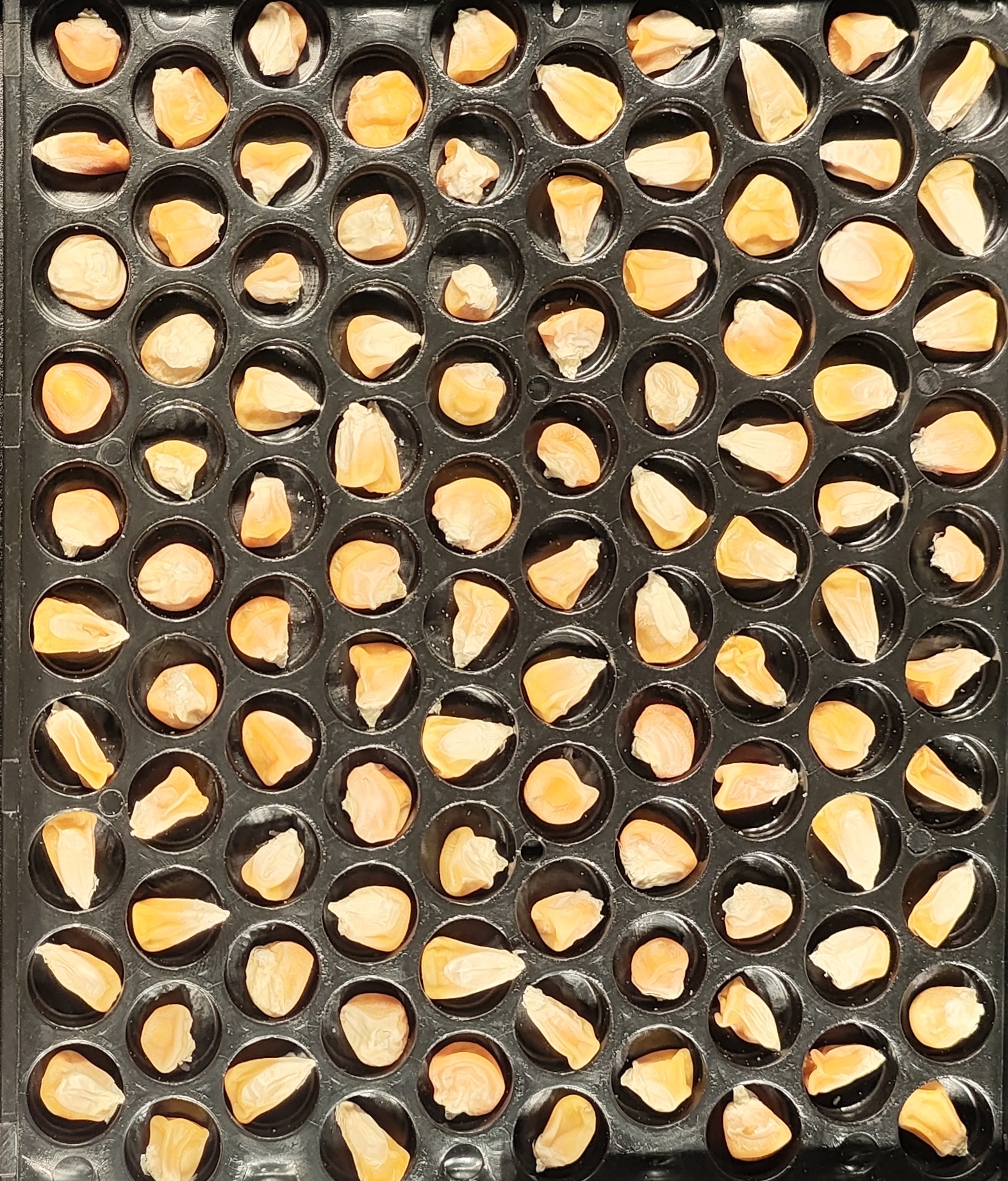

Supplement: Supplementary file 1 [file mmc1.zip › Spatial Registration/Jinxitian-1/Jinxitian_2_rgb_reg_high.jpg]

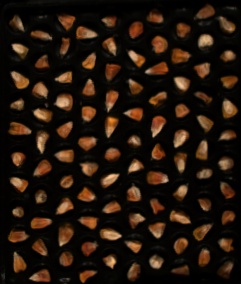

Supplement: Supplementary file 1 [file mmc1.zip › Spatial Registration/Jinxitian-2/Jinxitian_3_hsi_pseudo.jpg]

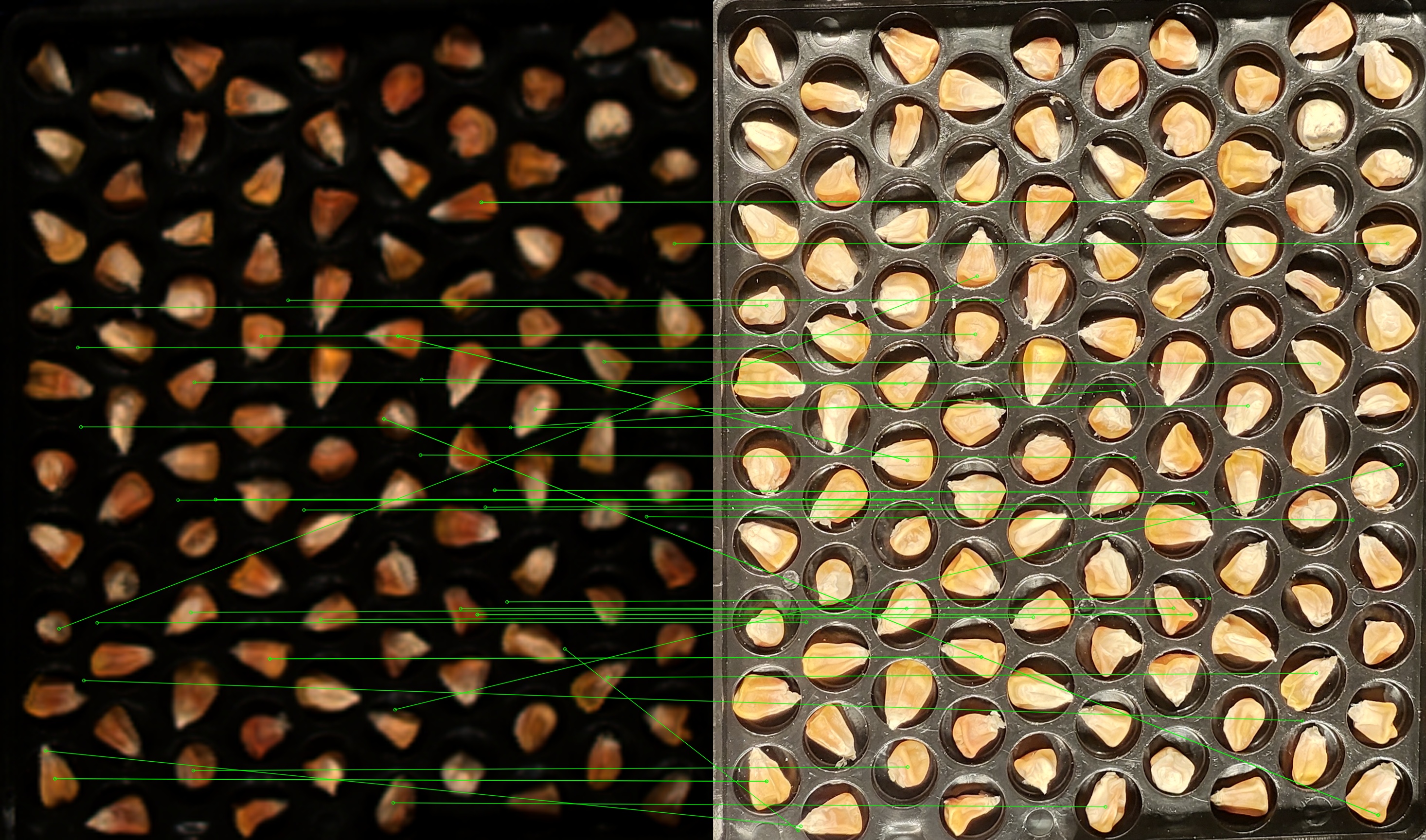

Supplement: Supplementary file 1 [file mmc1.zip › Spatial Registration/Jinxitian-2/Jinxitian_3_matches_viz.jpg]

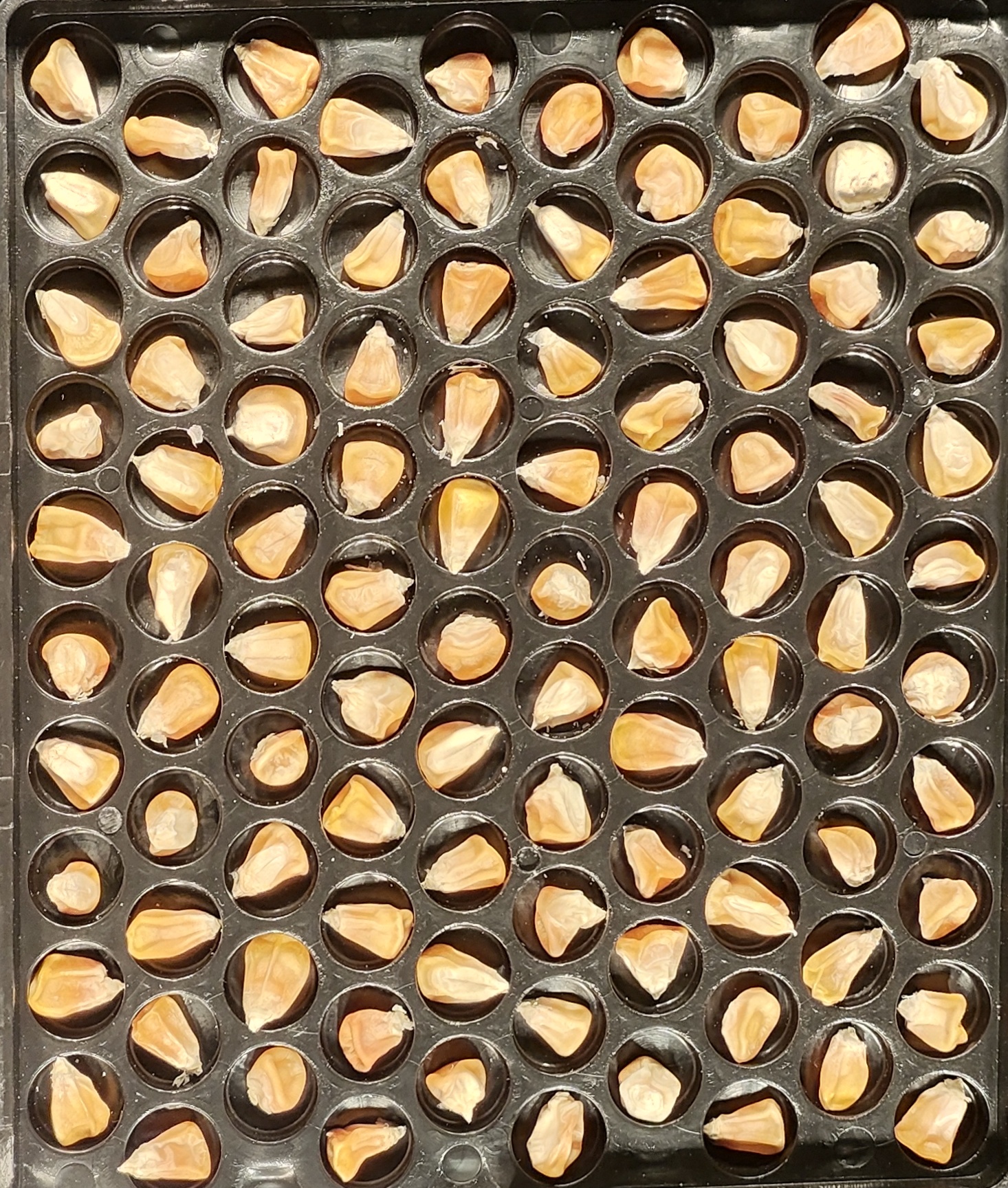

Supplement: Supplementary file 1 [file mmc1.zip › Spatial Registration/Jinxitian-2/Jinxitian_3_rgb_reg_high.jpg]

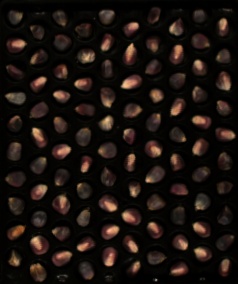

Supplement: Supplementary file 1 [file mmc1.zip › Spatial Registration/Mihuatiannuo No.3-1/Mihuatiannuo No.3_2_hsi_pseudo.jpg]

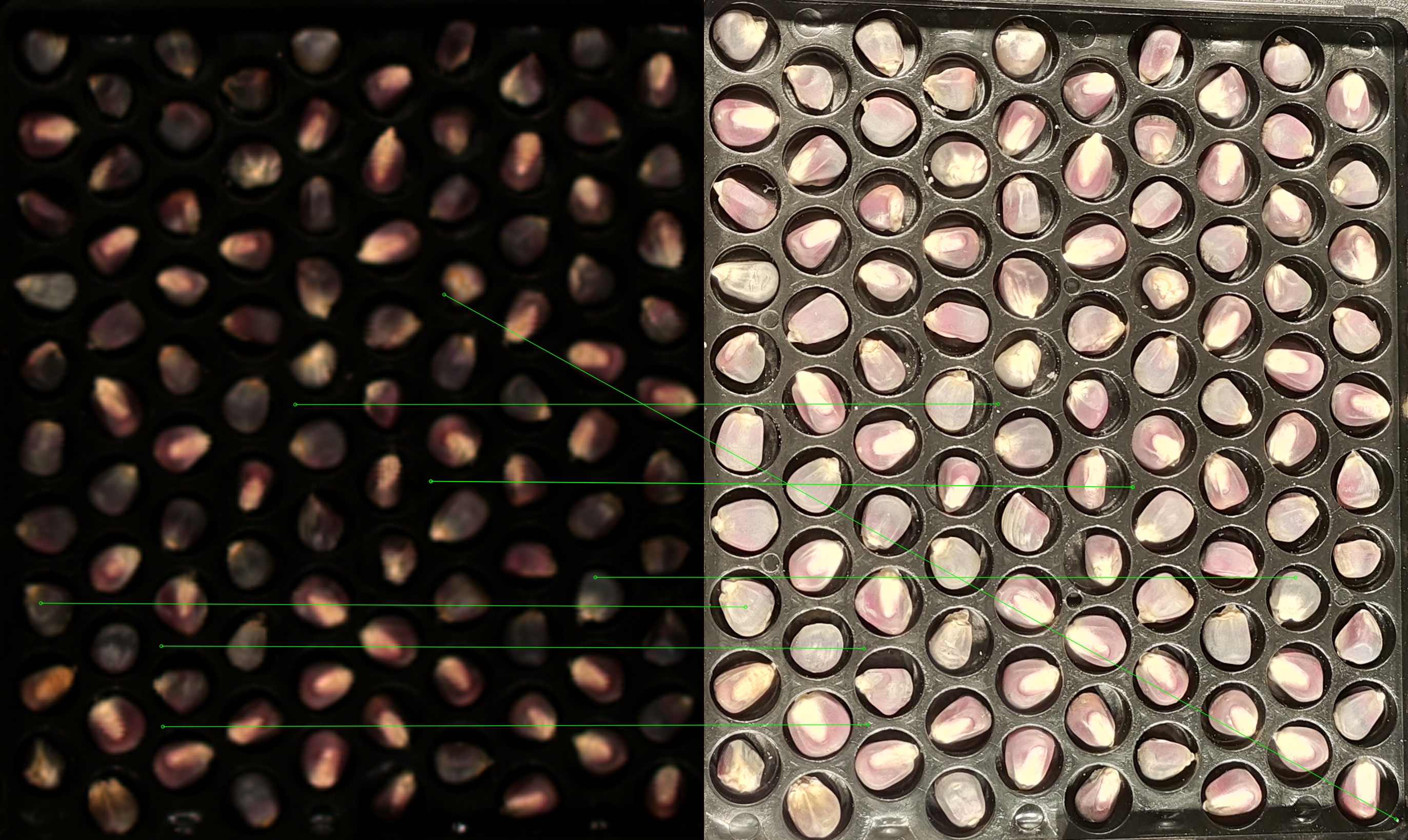

Supplement: Supplementary file 1 [file mmc1.zip › Spatial Registration/Mihuatiannuo No.3-1/Mihuatiannuo No.3_2_matches_viz.jpg]

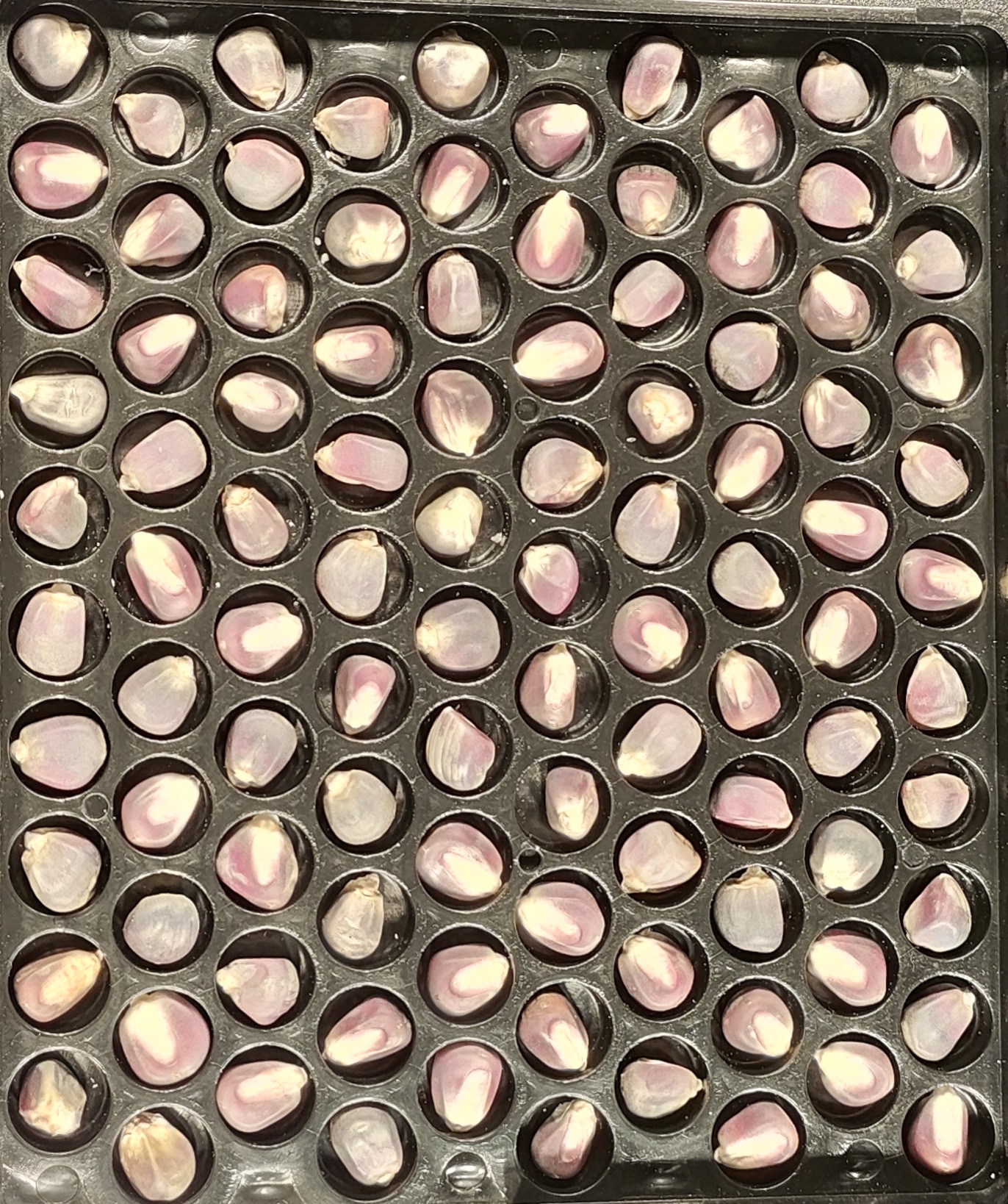

Supplement: Supplementary file 1 [file mmc1.zip › Spatial Registration/Mihuatiannuo No.3-1/Mihuatiannuo No.3_2_rgb_reg_high.jpg]

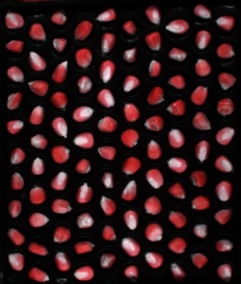

Supplement: Supplementary file 1 [file mmc1.zip › Spatial Registration/Mihuatiannuo No.3-2/test_hsi_pseudo.jpg]

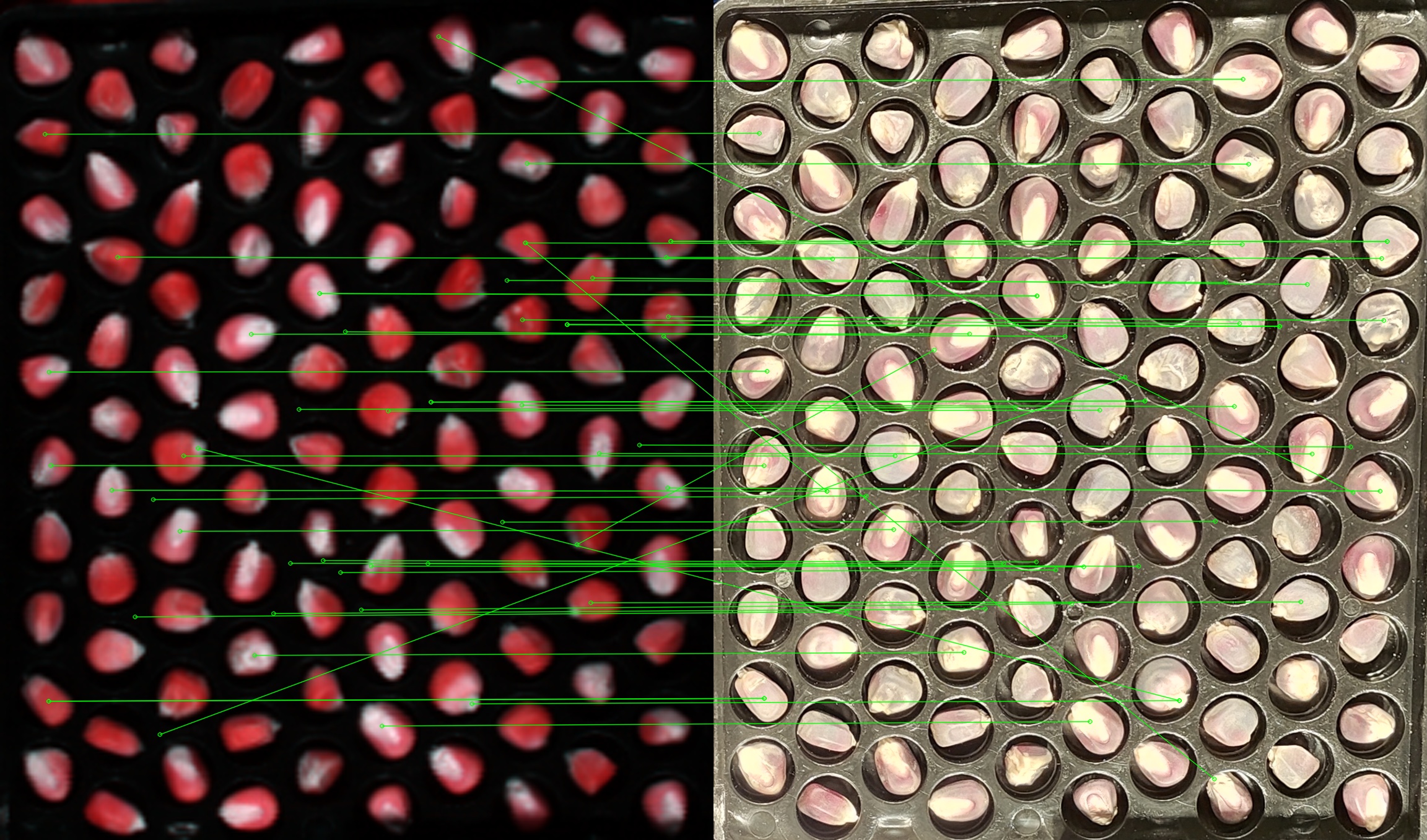

Supplement: Supplementary file 1 [file mmc1.zip › Spatial Registration/Mihuatiannuo No.3-2/test_matches_viz.jpg]

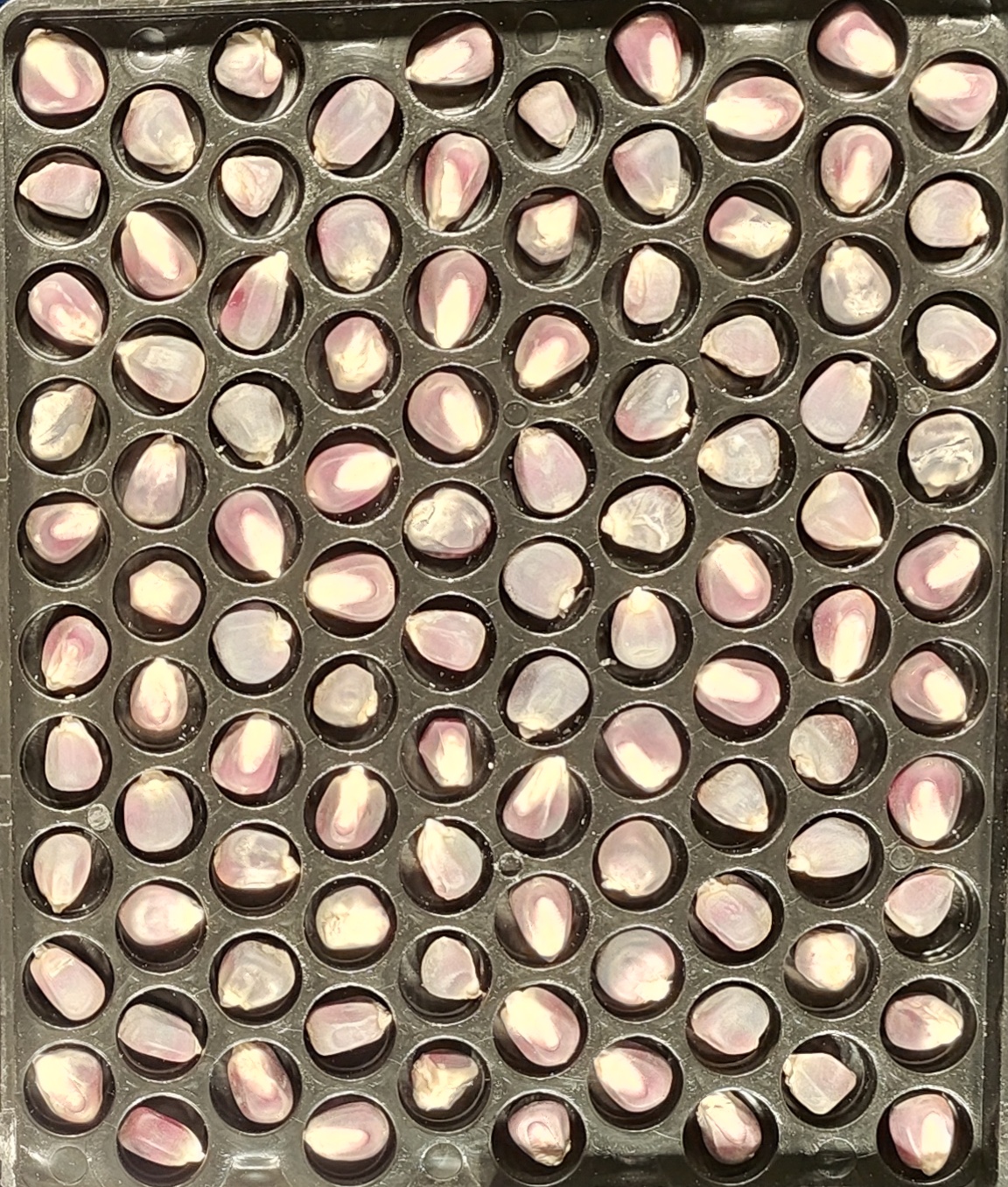

Supplement: Supplementary file 1 [file mmc1.zip › Spatial Registration/Mihuatiannuo No.3-2/test_rgb_reg_high.jpg]

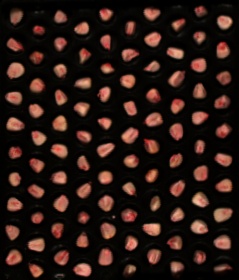

Supplement: Supplementary file 1 [file mmc1.zip › Spatial Registration/Mitiannuo No.1-1/Mitiannuo No.1_2_hsi_pseudo.jpg]

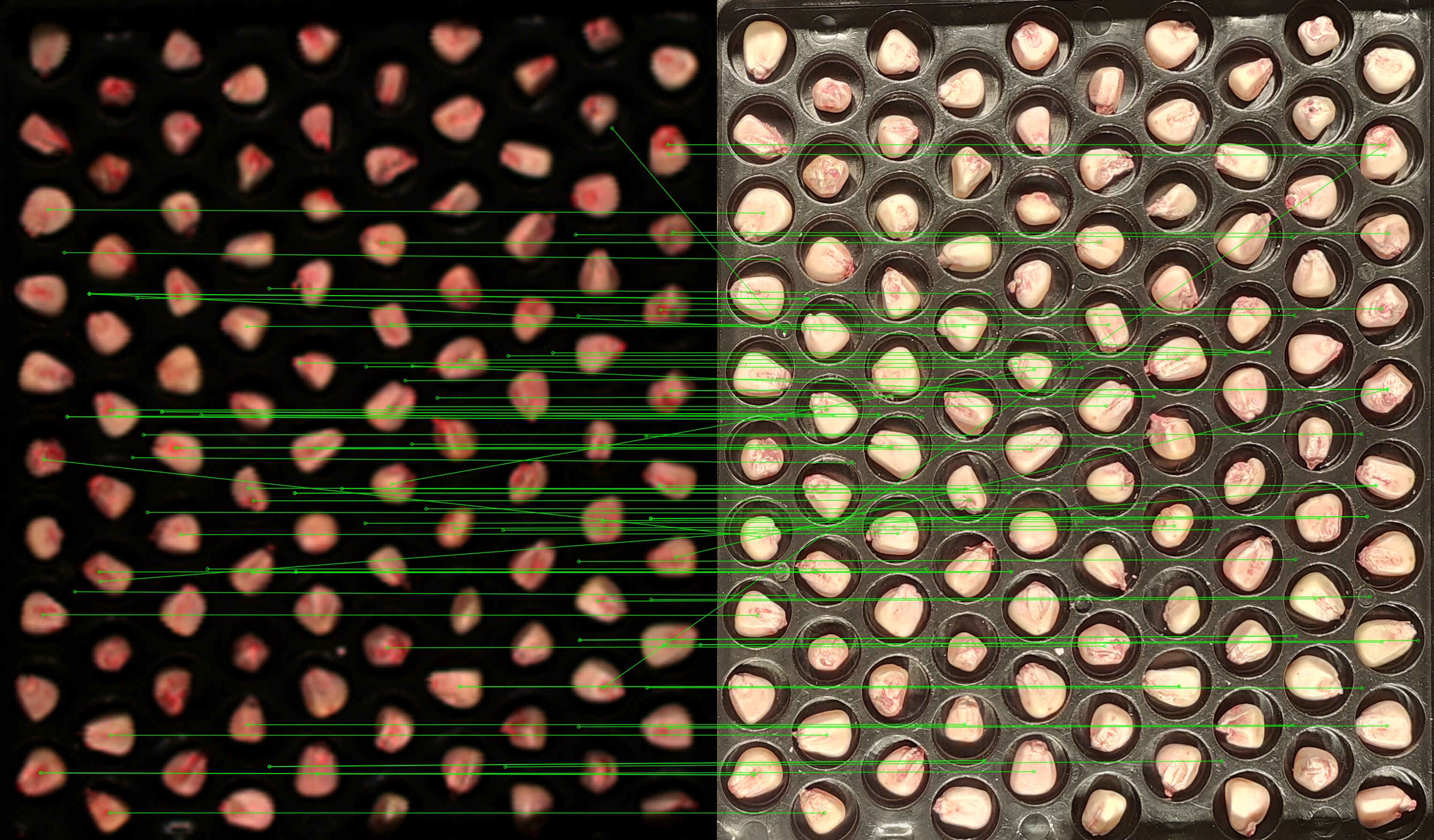

Supplement: Supplementary file 1 [file mmc1.zip › Spatial Registration/Mitiannuo No.1-1/Mitiannuo No.1_2_matches_viz.jpg]

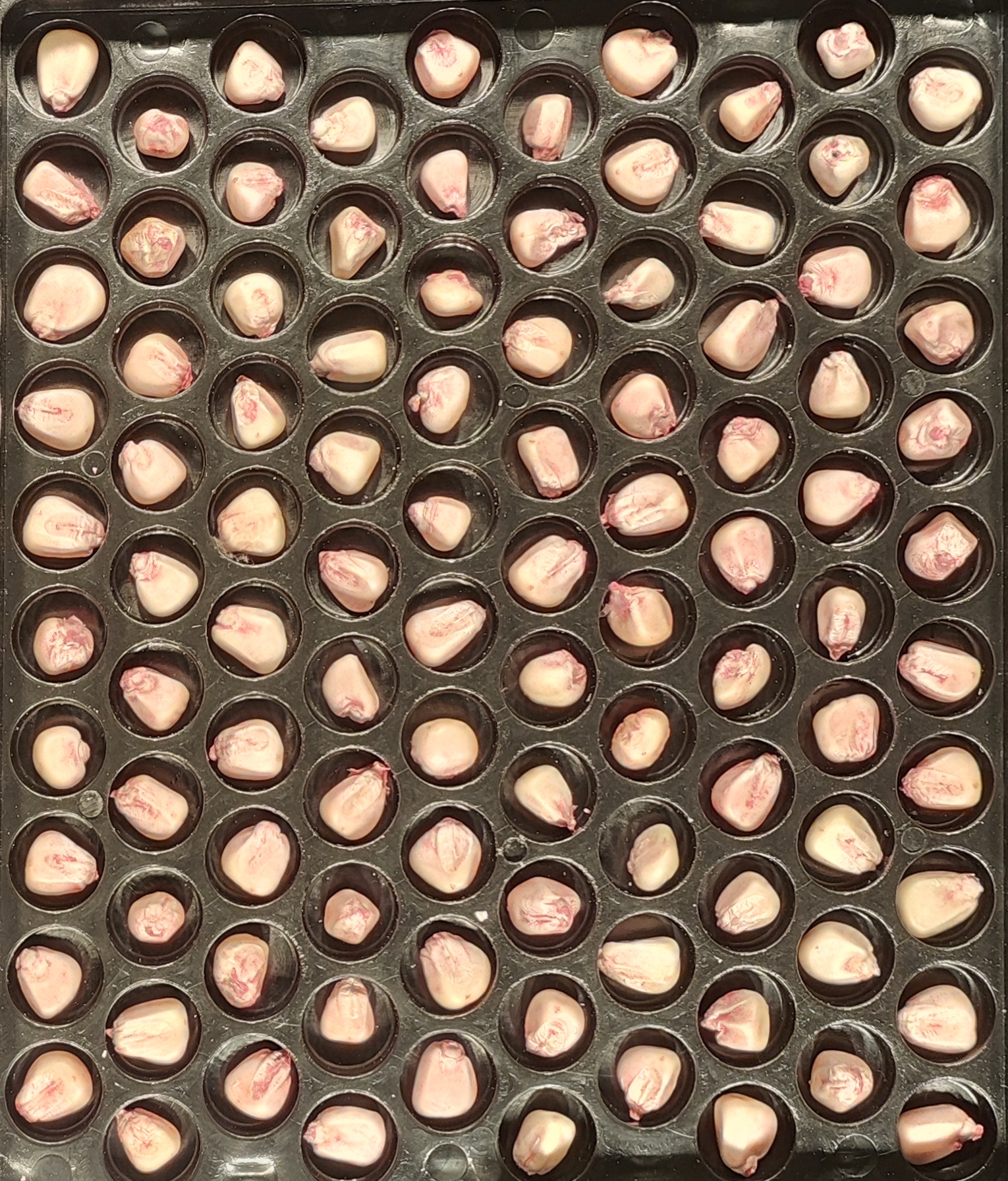

Supplement: Supplementary file 1 [file mmc1.zip › Spatial Registration/Mitiannuo No.1-1/Mitiannuo No.1_2_rgb_reg_high.jpg]

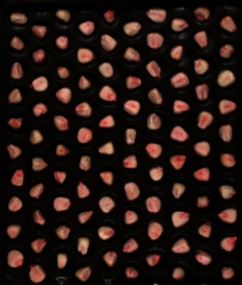

Supplement: Supplementary file 1 [file mmc1.zip › Spatial Registration/Mitiannuo No.1-2/Mitiannuo No.1_3_hsi_pseudo.jpg]

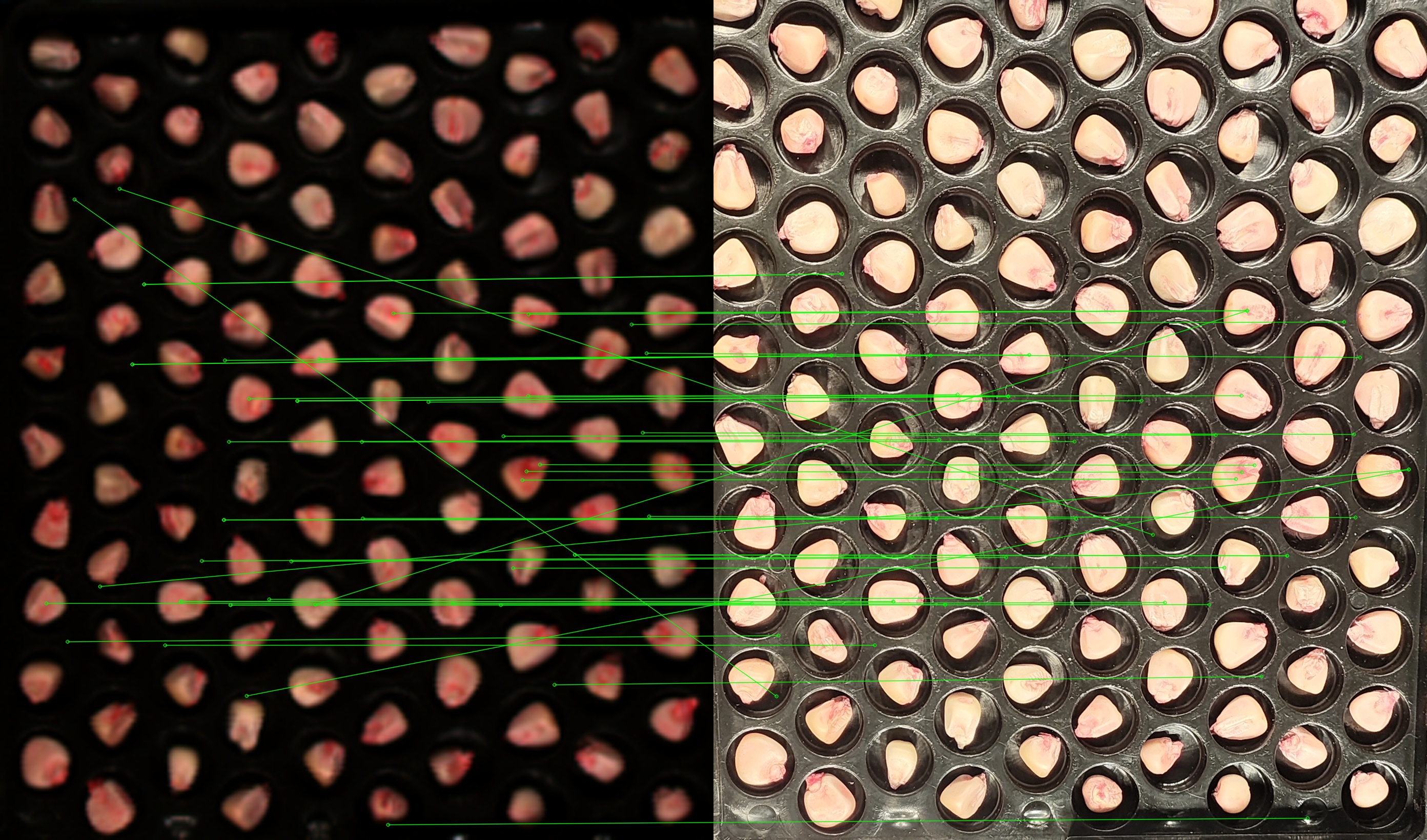

Supplement: Supplementary file 1 [file mmc1.zip › Spatial Registration/Mitiannuo No.1-2/Mitiannuo No.1_3_matches_viz.jpg]

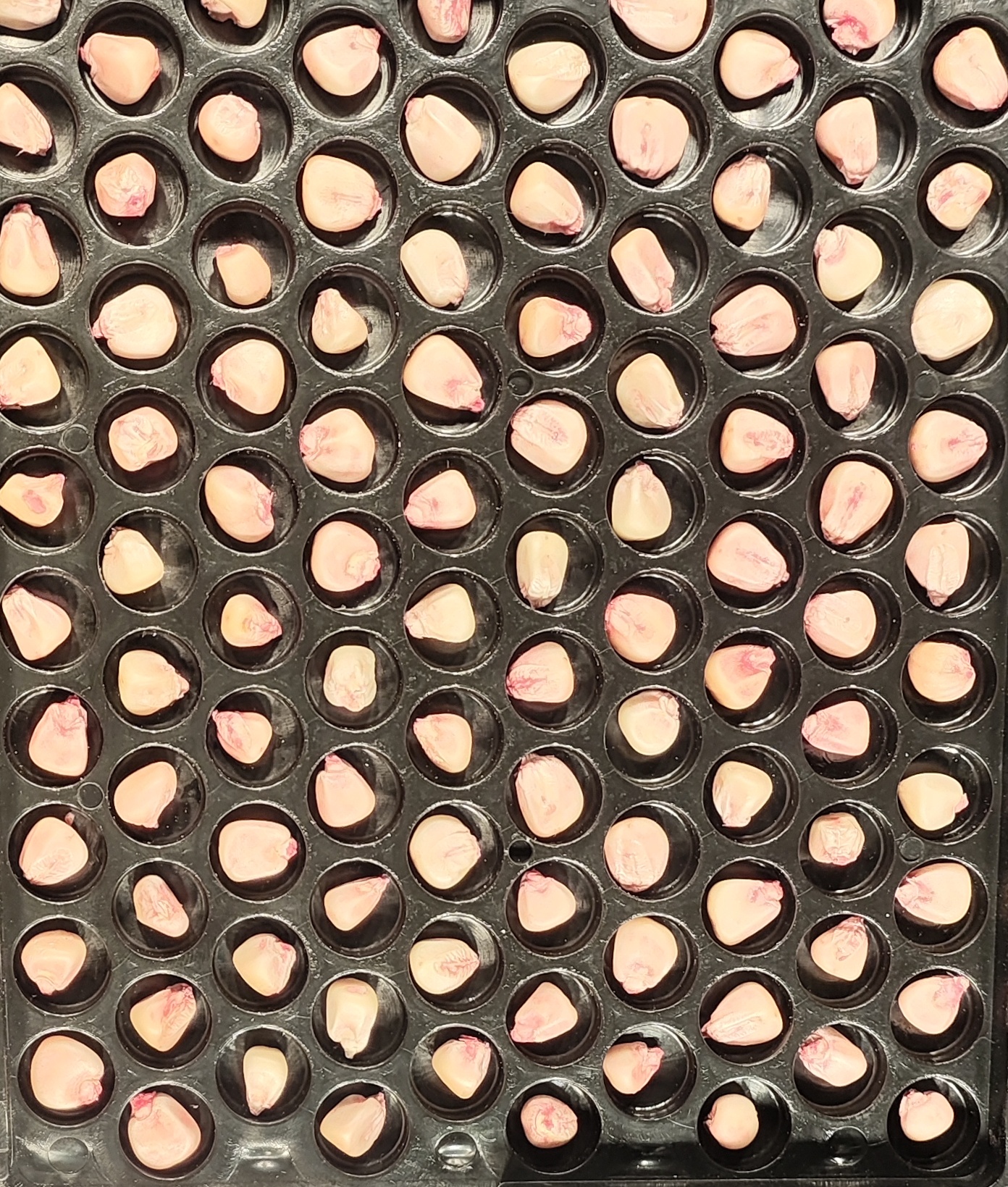

Supplement: Supplementary file 1 [file mmc1.zip › Spatial Registration/Mitiannuo No.1-2/Mitiannuo No.1_3_rgb_reg_high.jpg]

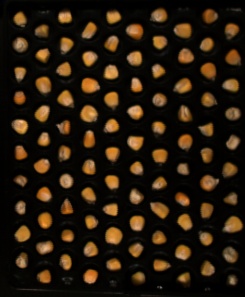

Supplement: Supplementary file 1 [file mmc1.zip › Spatial Registration/Mitiannuo No.4-1/Mitiannuo No.4_2_hsi_pseudo.jpg]

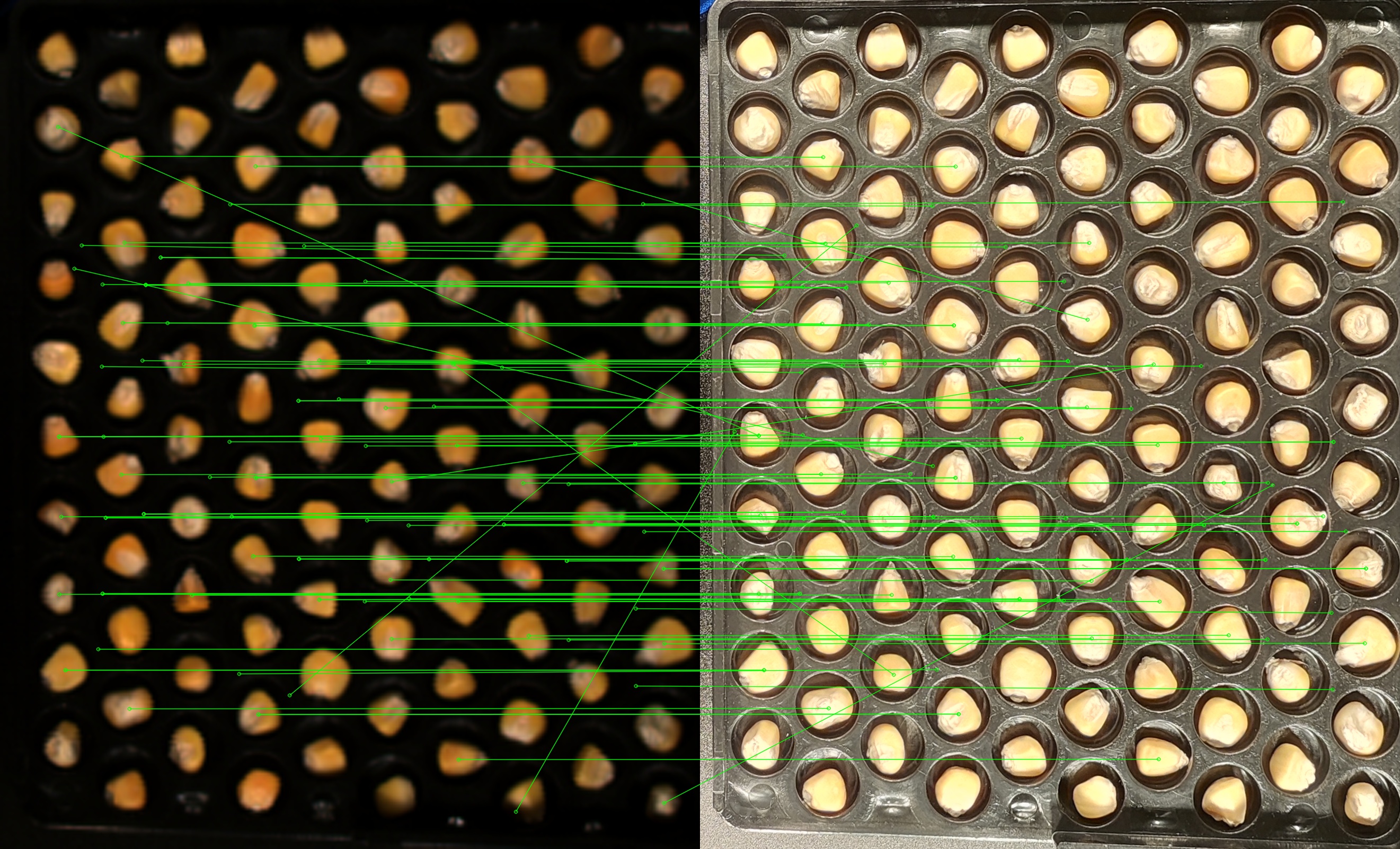

Supplement: Supplementary file 1 [file mmc1.zip › Spatial Registration/Mitiannuo No.4-1/Mitiannuo No.4_2_matches_viz.jpg]

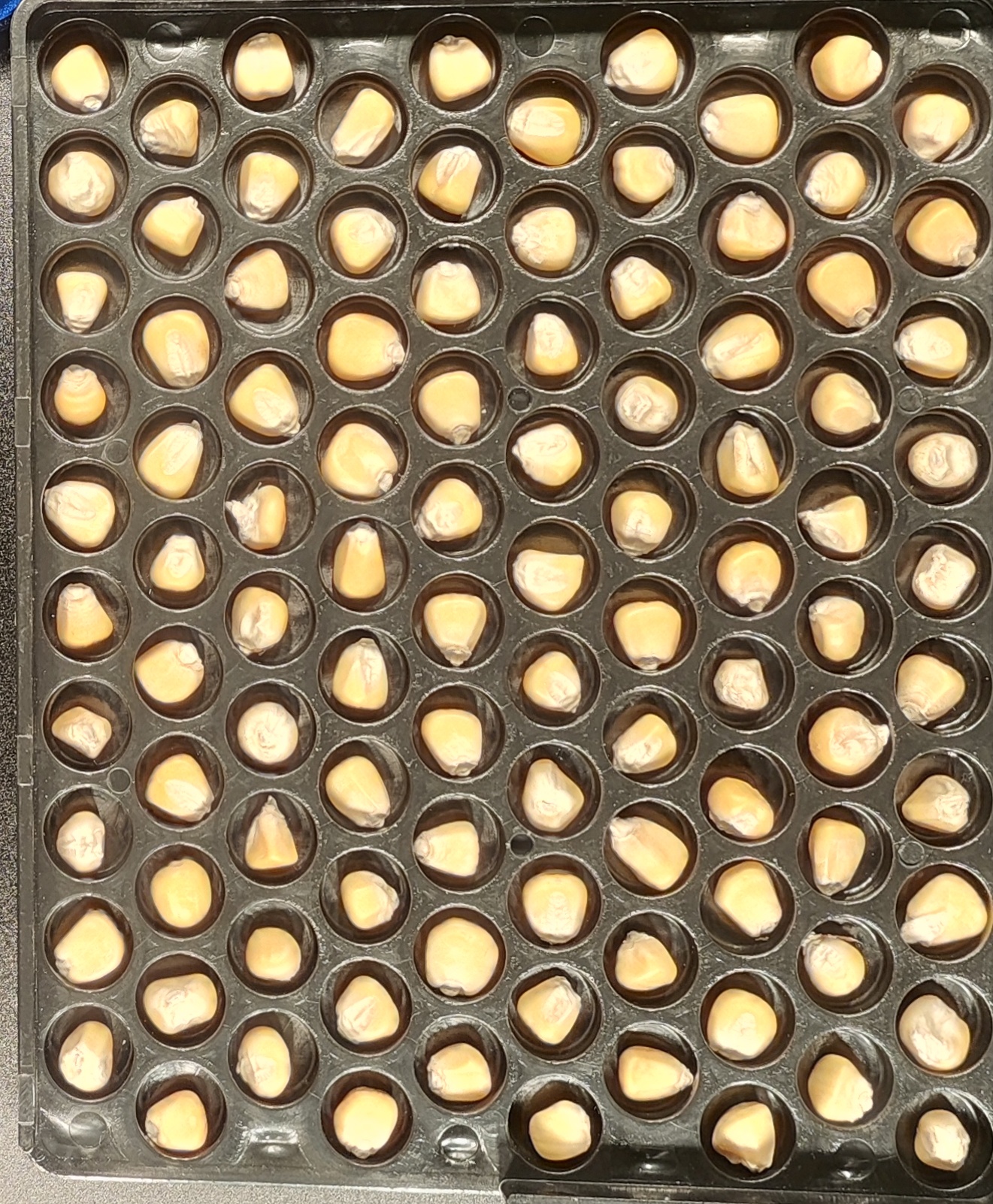

Supplement: Supplementary file 1 [file mmc1.zip › Spatial Registration/Mitiannuo No.4-1/Mitiannuo No.4_2_rgb_reg_high.jpg]

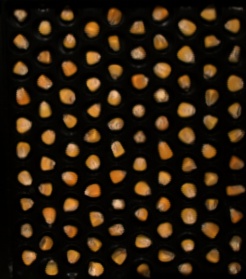

Supplement: Supplementary file 1 [file mmc1.zip › Spatial Registration/Mitiannuo No.4-2/Mitiannuo No.4_3_hsi_pseudo.jpg]

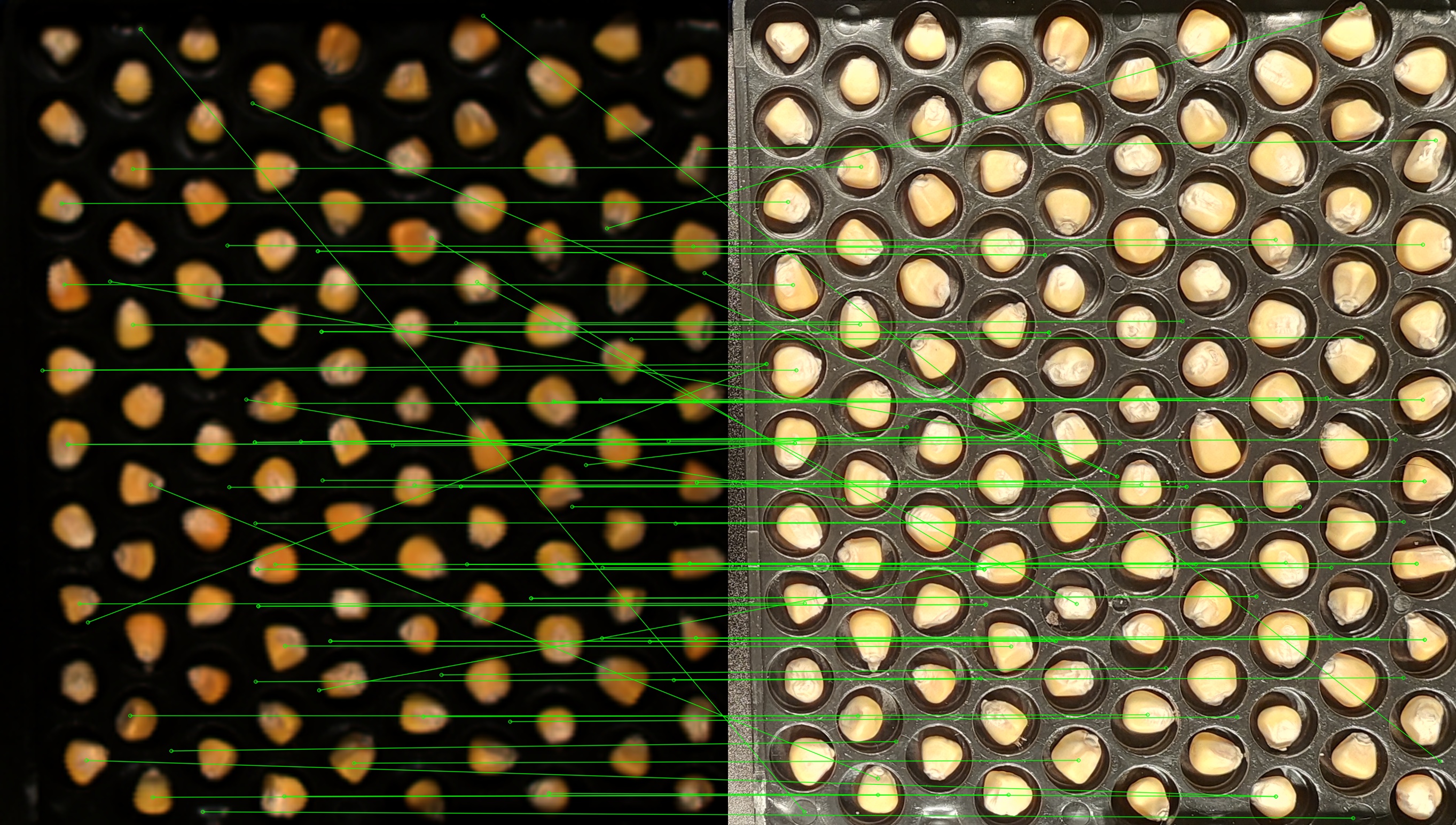

Supplement: Supplementary file 1 [file mmc1.zip › Spatial Registration/Mitiannuo No.4-2/Mitiannuo No.4_3_matches_viz.jpg]

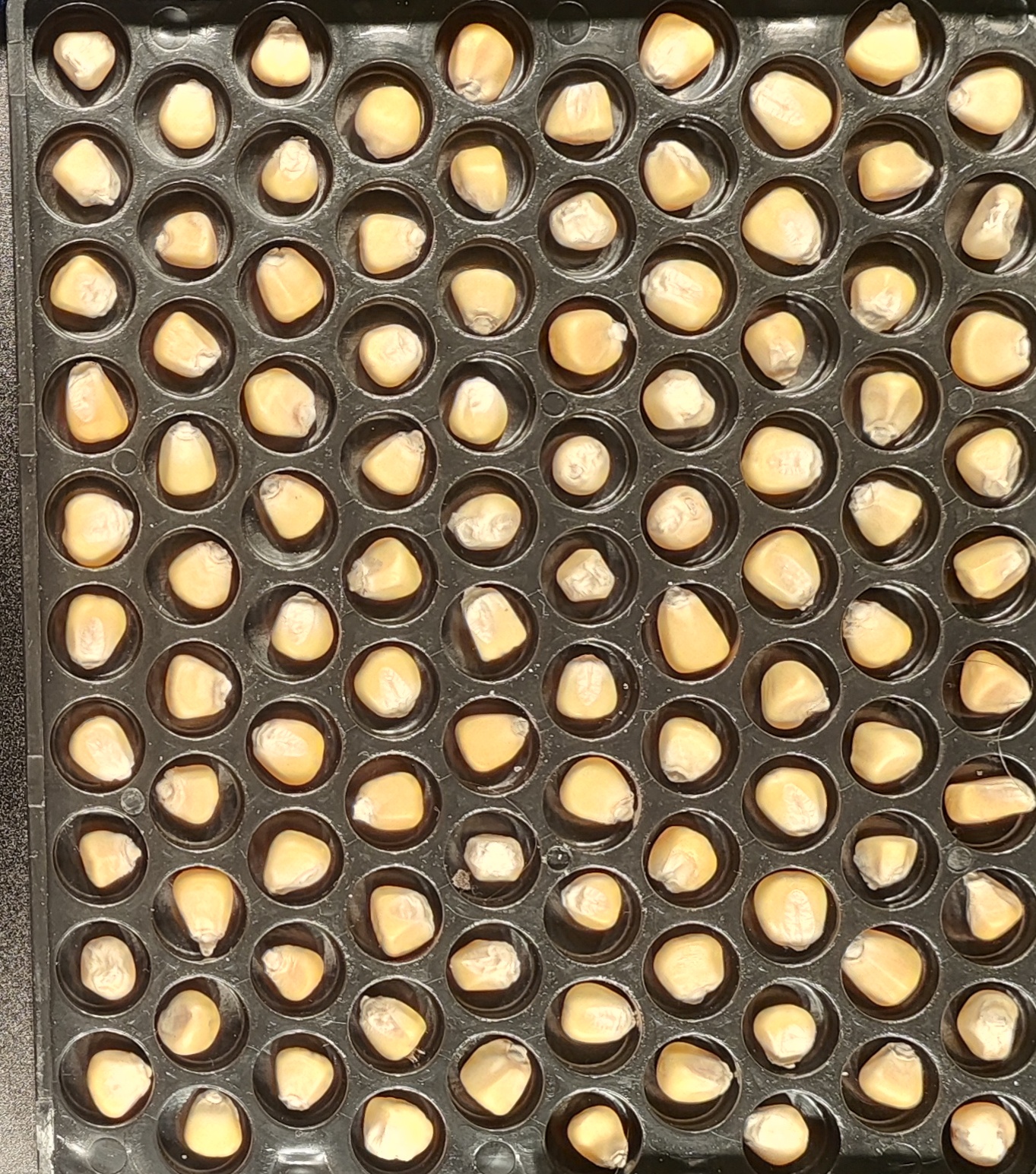

Supplement: Supplementary file 1 [file mmc1.zip › Spatial Registration/Mitiannuo No.4-2/Mitiannuo No.4_3_rgb_reg_high.jpg]

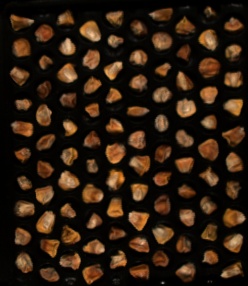

Supplement: Supplementary file 1 [file mmc1.zip › Spatial Registration/Sida No.4-1/Sida No.4_2_hsi_pseudo.jpg]

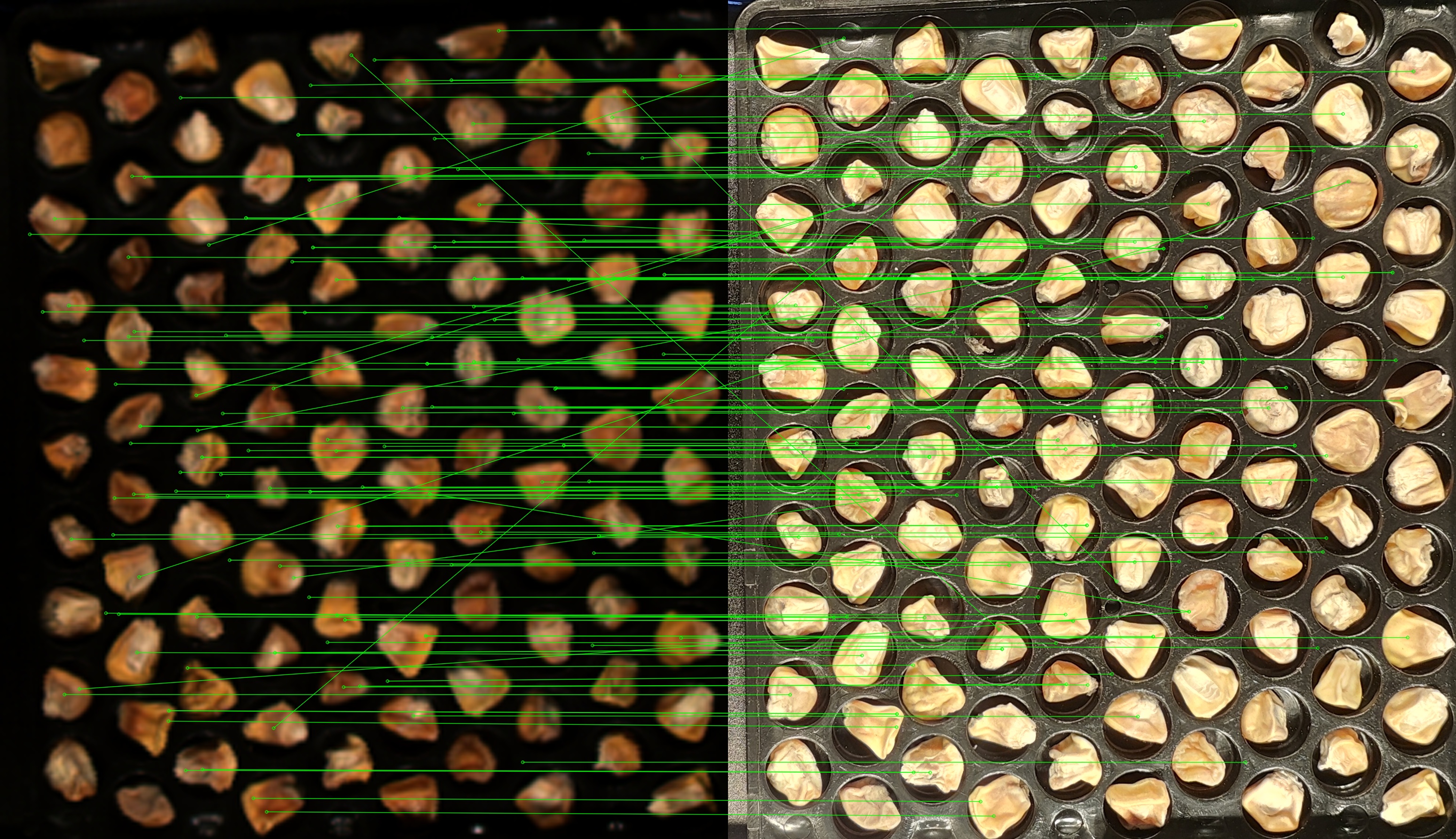

Supplement: Supplementary file 1 [file mmc1.zip › Spatial Registration/Sida No.4-1/Sida No.4_2_matches_viz.jpg]

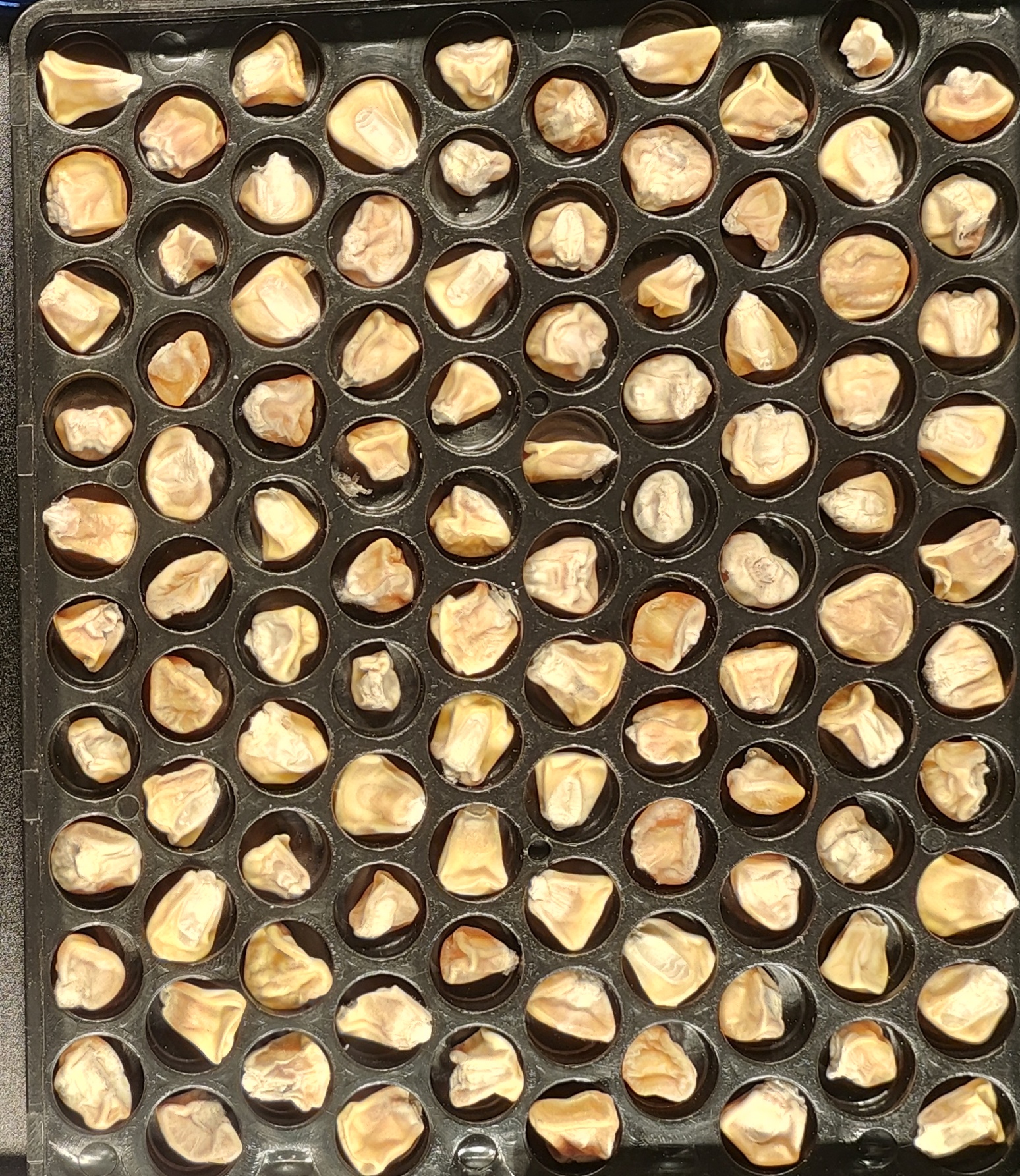

Supplement: Supplementary file 1 [file mmc1.zip › Spatial Registration/Sida No.4-1/Sida No.4_2_rgb_reg_high.jpg]

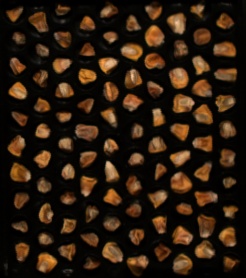

Supplement: Supplementary file 1 [file mmc1.zip › Spatial Registration/Sida No.4-2/Sida No.4_3_hsi_pseudo.jpg]

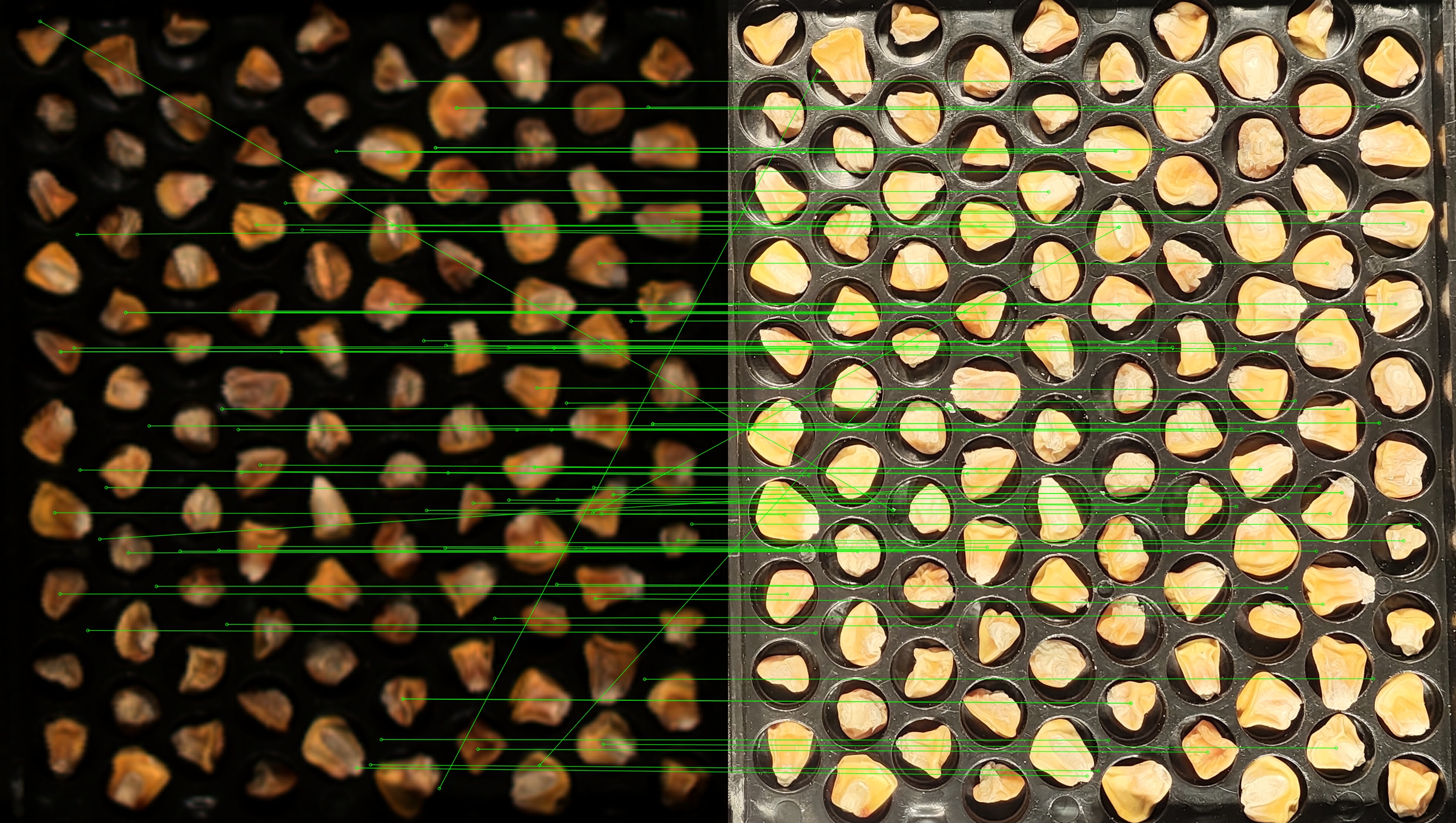

Supplement: Supplementary file 1 [file mmc1.zip › Spatial Registration/Sida No.4-2/Sida No.4_3_matches_viz.jpg]

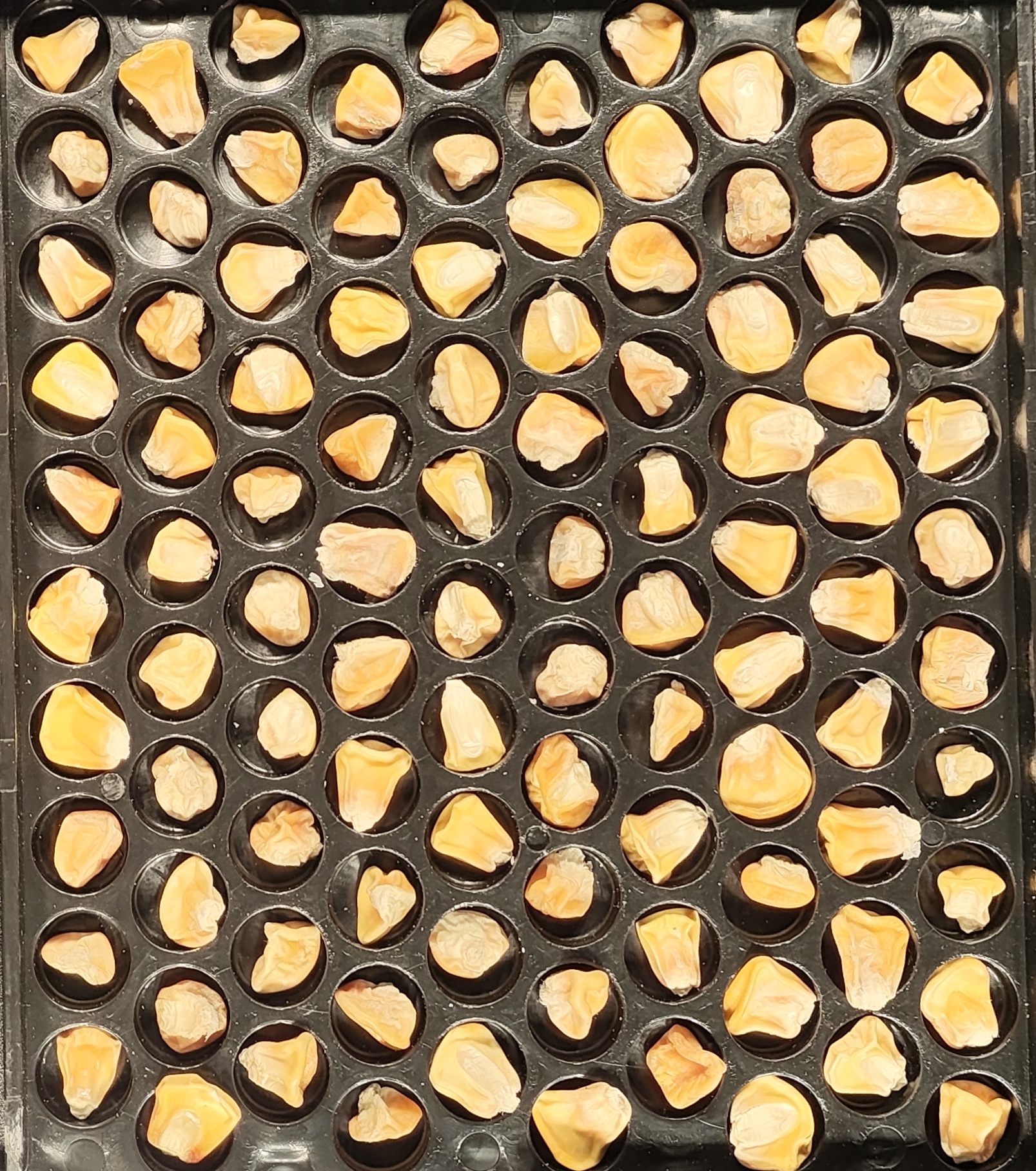

Supplement: Supplementary file 1 [file mmc1.zip › Spatial Registration/Sida No.4-2/Sida No.4_3_rgb_reg_high.jpg]

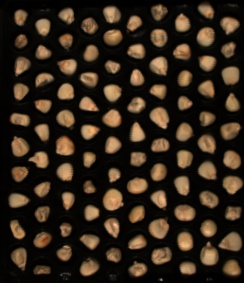

Supplement: Supplementary file 1 [file mmc1.zip › Spatial Registration/Sidanuo No.38-1/Sidanuo No.38_2_hsi_pseudo.jpg]

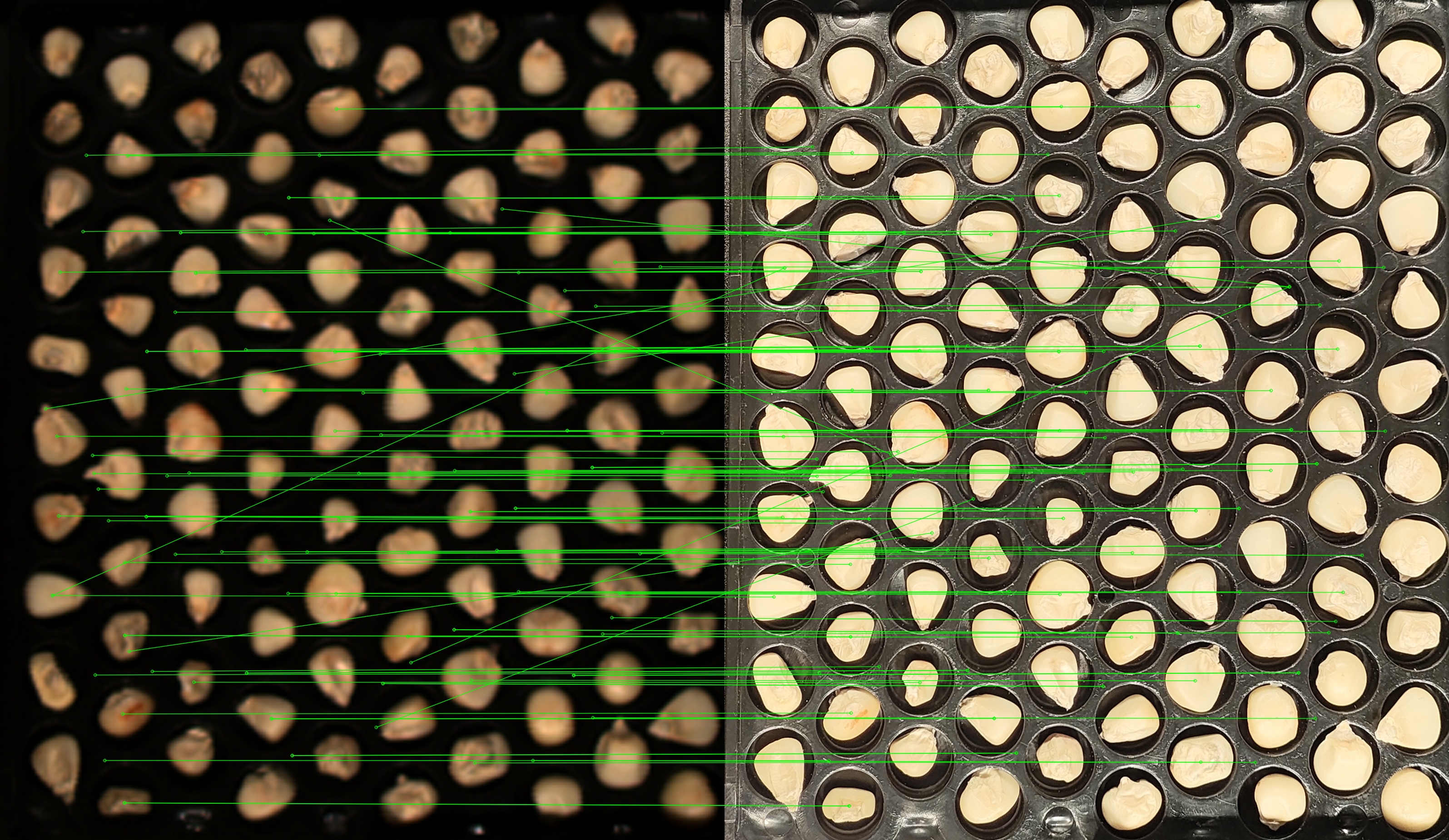

Supplement: Supplementary file 1 [file mmc1.zip › Spatial Registration/Sidanuo No.38-1/Sidanuo No.38_2_matches_viz.jpg]

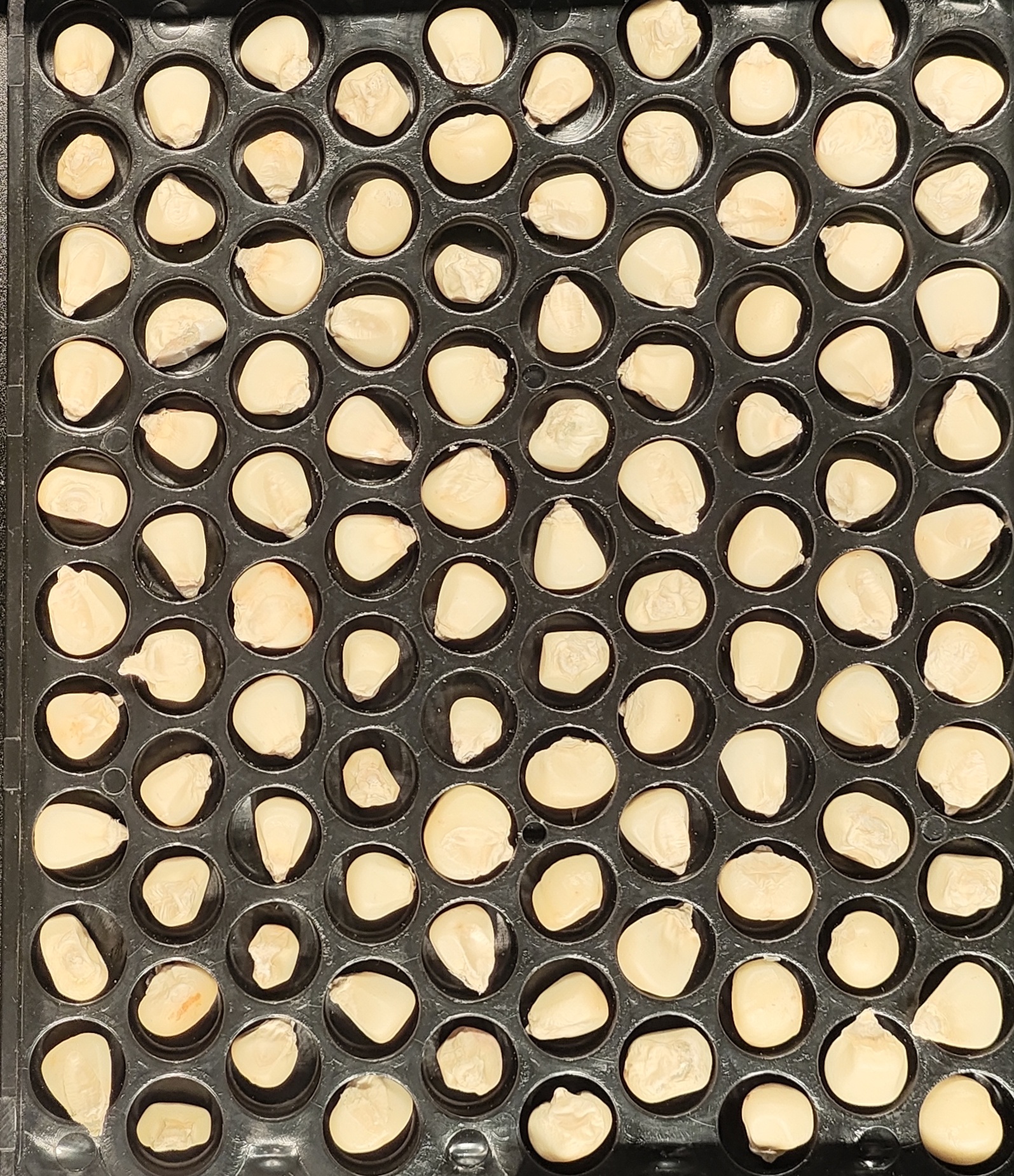

Supplement: Supplementary file 1 [file mmc1.zip › Spatial Registration/Sidanuo No.38-1/Sidanuo No.38_2_rgb_reg_high.jpg]

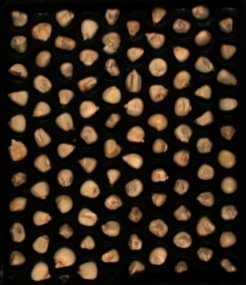

Supplement: Supplementary file 1 [file mmc1.zip › Spatial Registration/Sidanuo No.38-2/Sidanuo No.38_3_hsi_pseudo.jpg]

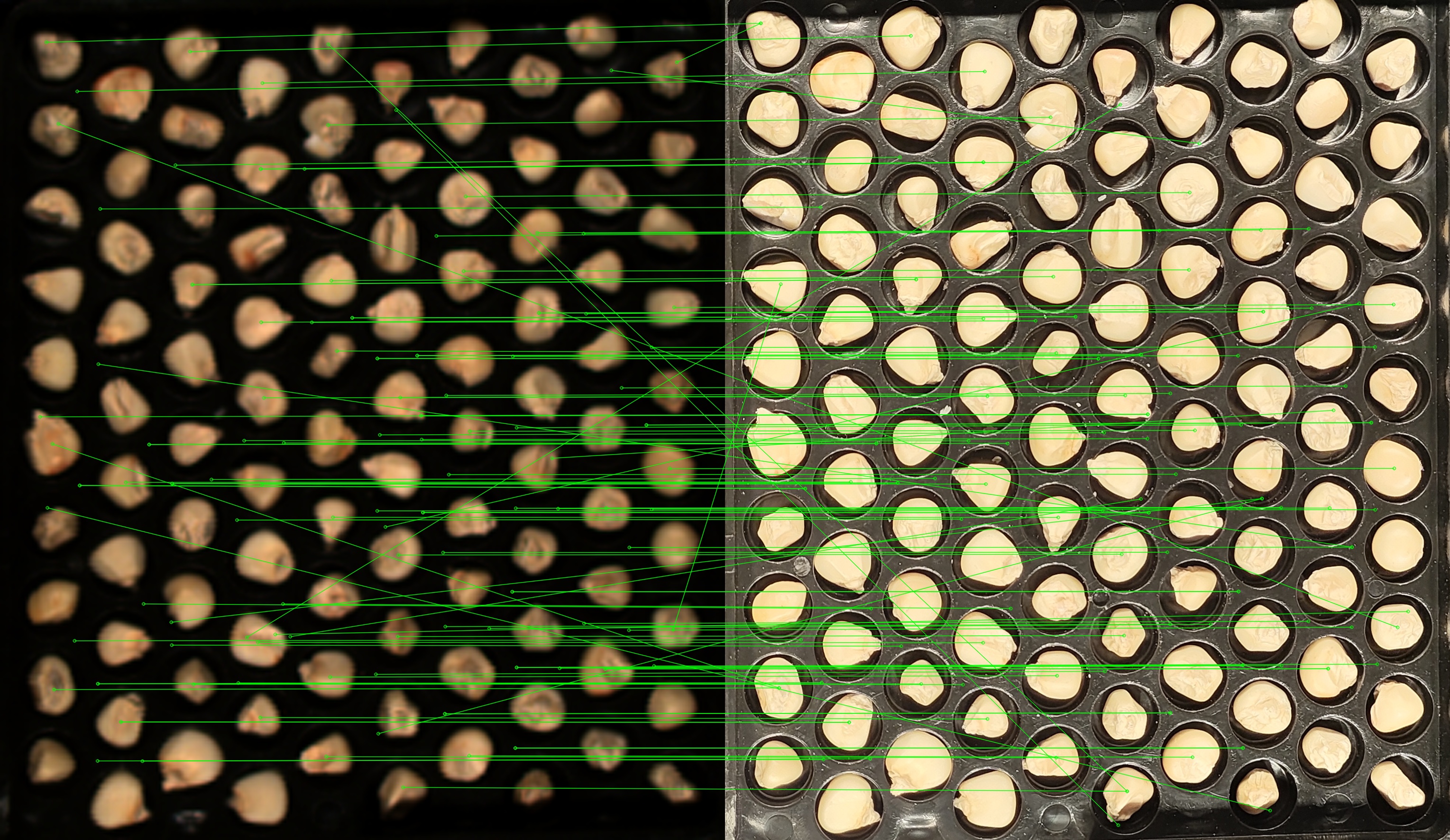

Supplement: Supplementary file 1 [file mmc1.zip › Spatial Registration/Sidanuo No.38-2/Sidanuo No.38_3_matches_viz.jpg]

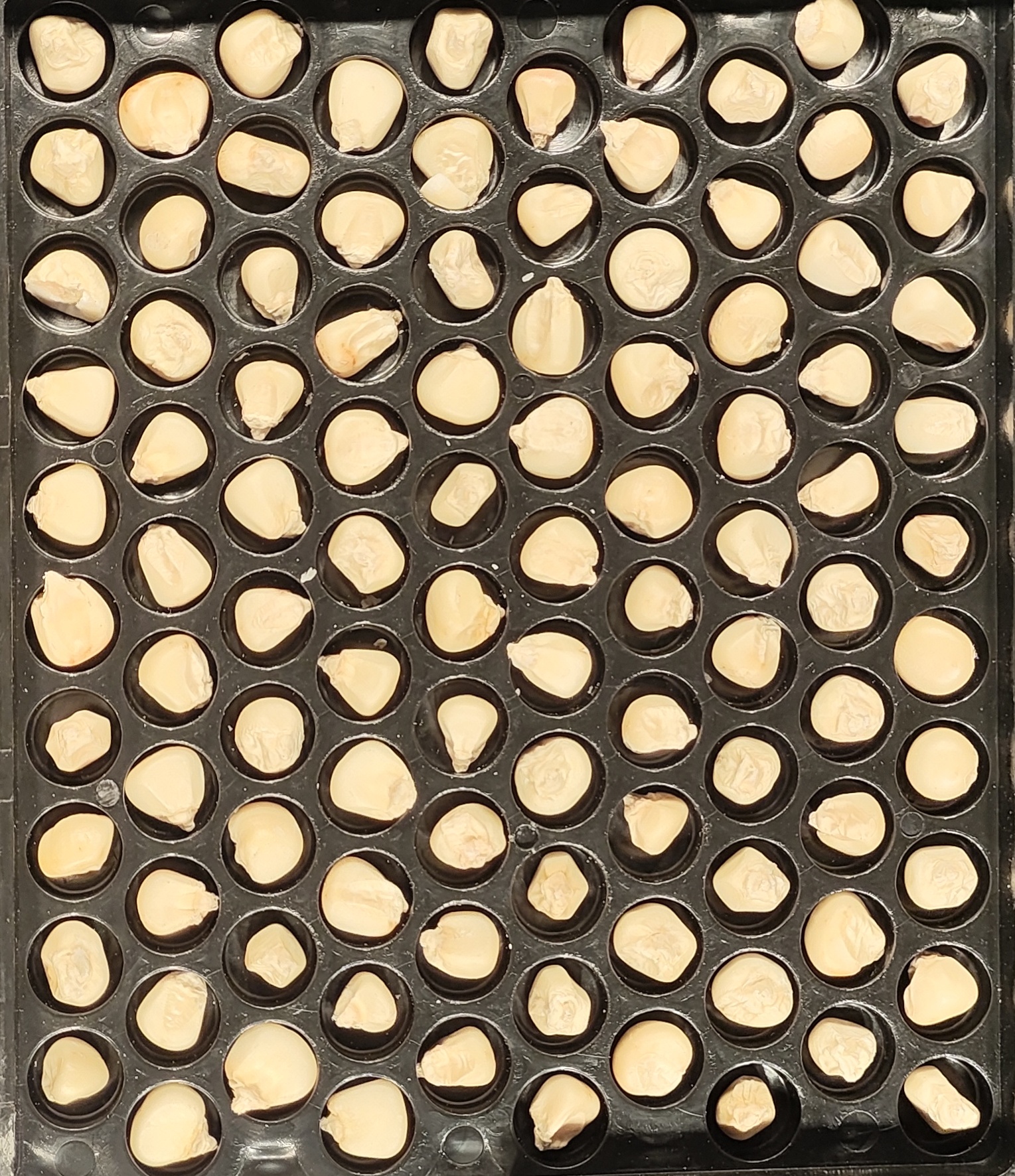

Supplement: Supplementary file 1 [file mmc1.zip › Spatial Registration/Sidanuo No.38-2/Sidanuo No.38_3_rgb_reg_high.jpg]

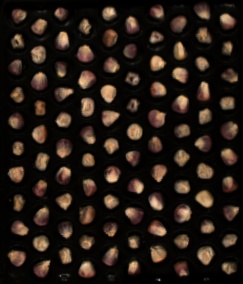

Supplement: Supplementary file 1 [file mmc1.zip › Spatial Registration/Sidanuo No.44-1/Sidanuo No.44_2_hsi_pseudo.jpg]

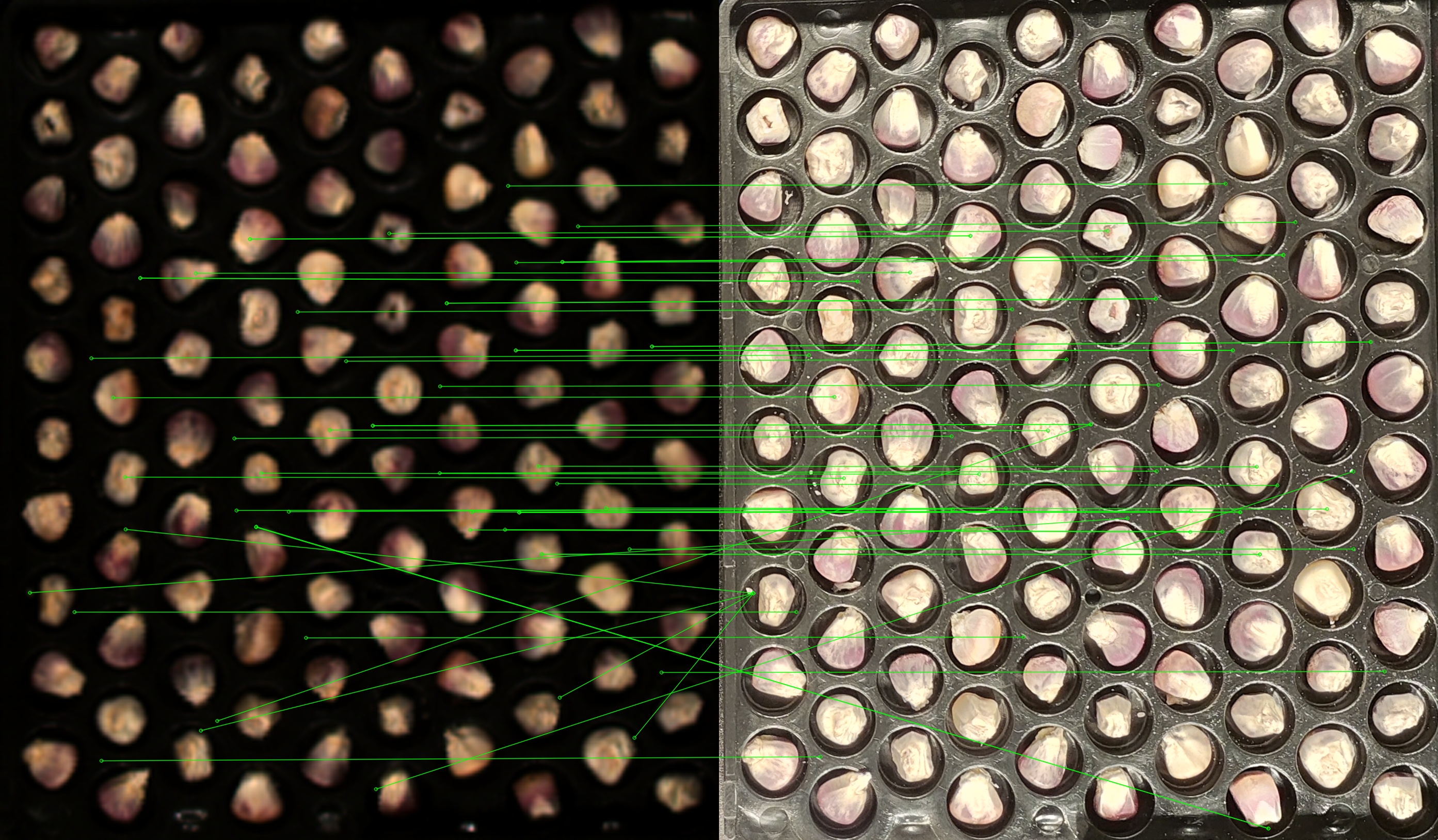

Supplement: Supplementary file 1 [file mmc1.zip › Spatial Registration/Sidanuo No.44-1/Sidanuo No.44_2_matches_viz.jpg]

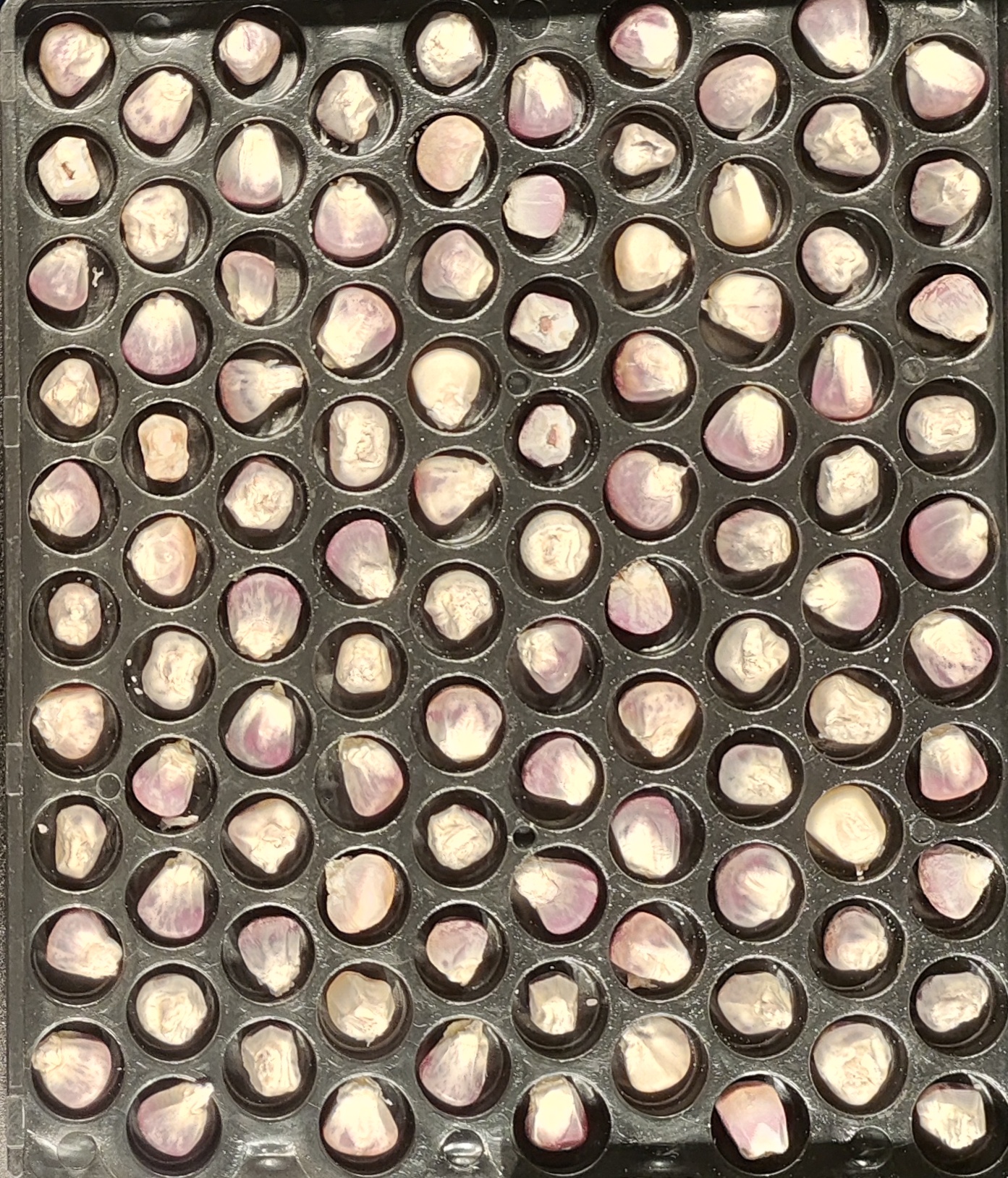

Supplement: Supplementary file 1 [file mmc1.zip › Spatial Registration/Sidanuo No.44-1/Sidanuo No.44_2_rgb_reg_high.jpg]

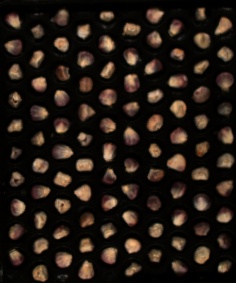

Supplement: Supplementary file 1 [file mmc1.zip › Spatial Registration/Sidanuo No.44-2/Sidanuo No.44_3_hsi_pseudo.jpg]

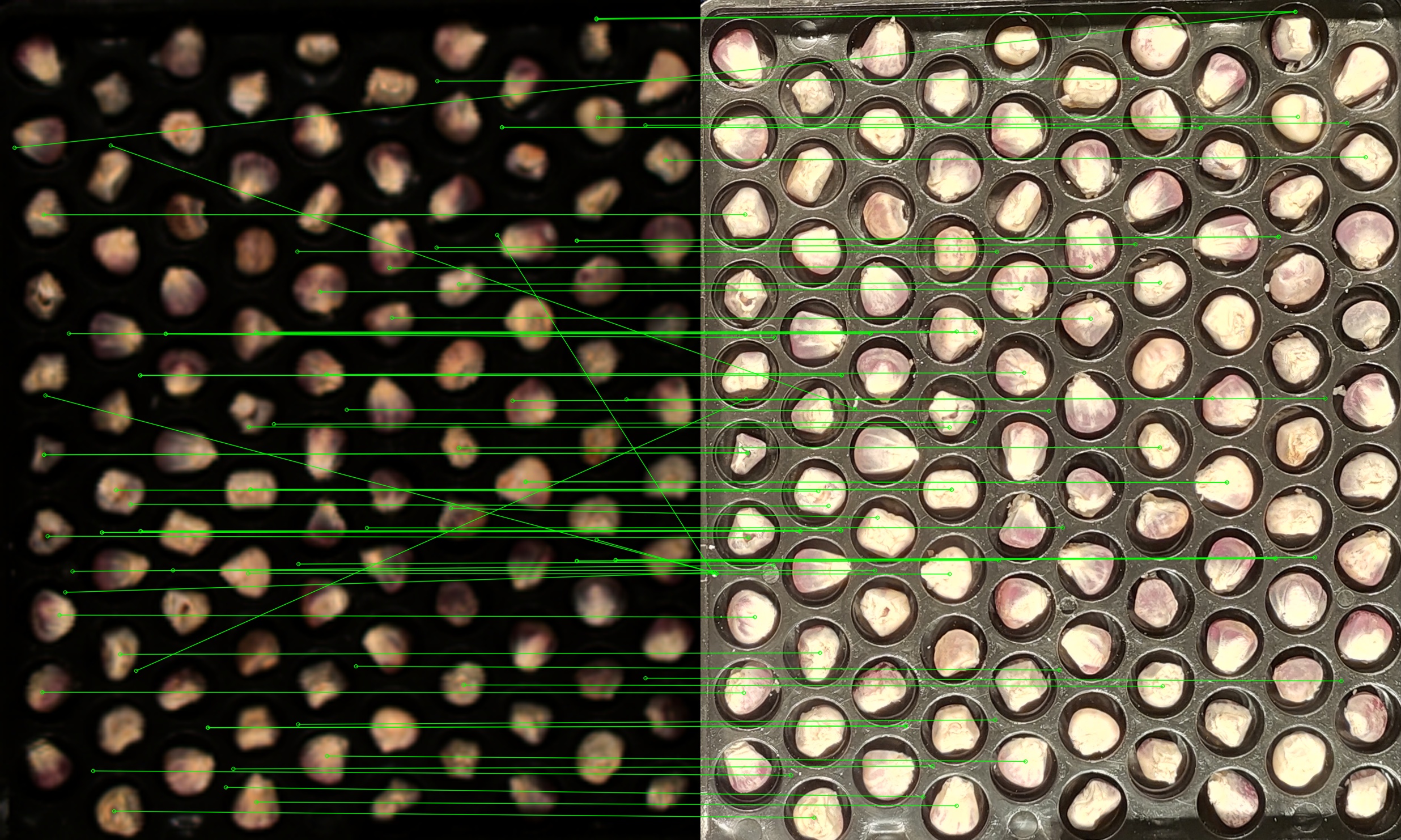

Supplement: Supplementary file 1 [file mmc1.zip › Spatial Registration/Sidanuo No.44-2/Sidanuo No.44_3_matches_viz.jpg]

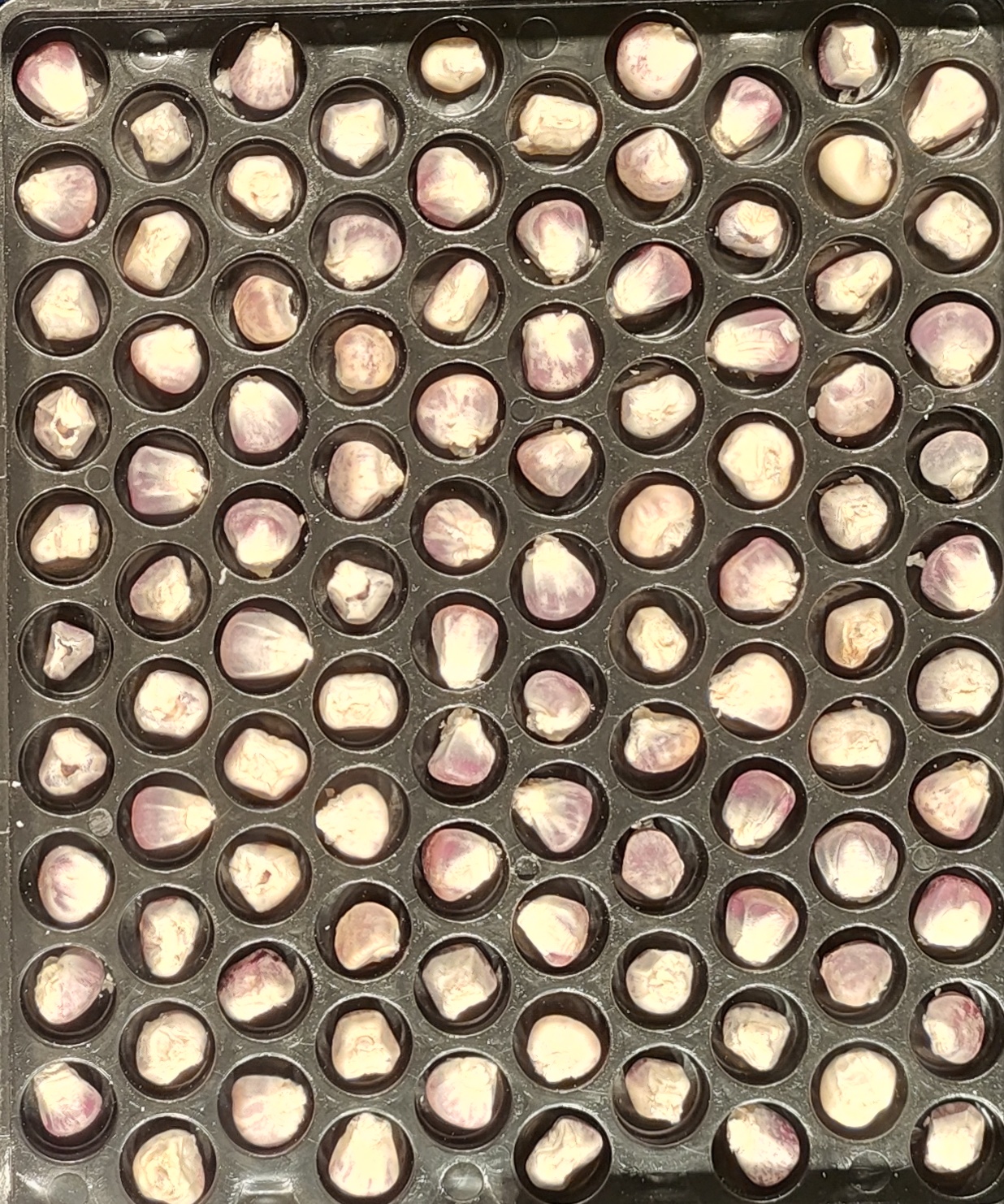

Supplement: Supplementary file 1 [file mmc1.zip › Spatial Registration/Sidanuo No.44-2/Sidanuo No.44_3_rgb_reg_high.jpg]

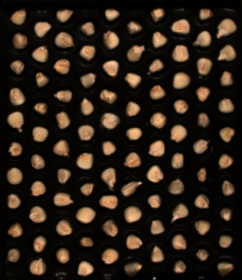

Supplement: Supplementary file 1 [file mmc1.zip › Spatial Registration/Zhuyunuo No.1-1/Zhuyunuo No.1_2_hsi_pseudo.jpg]

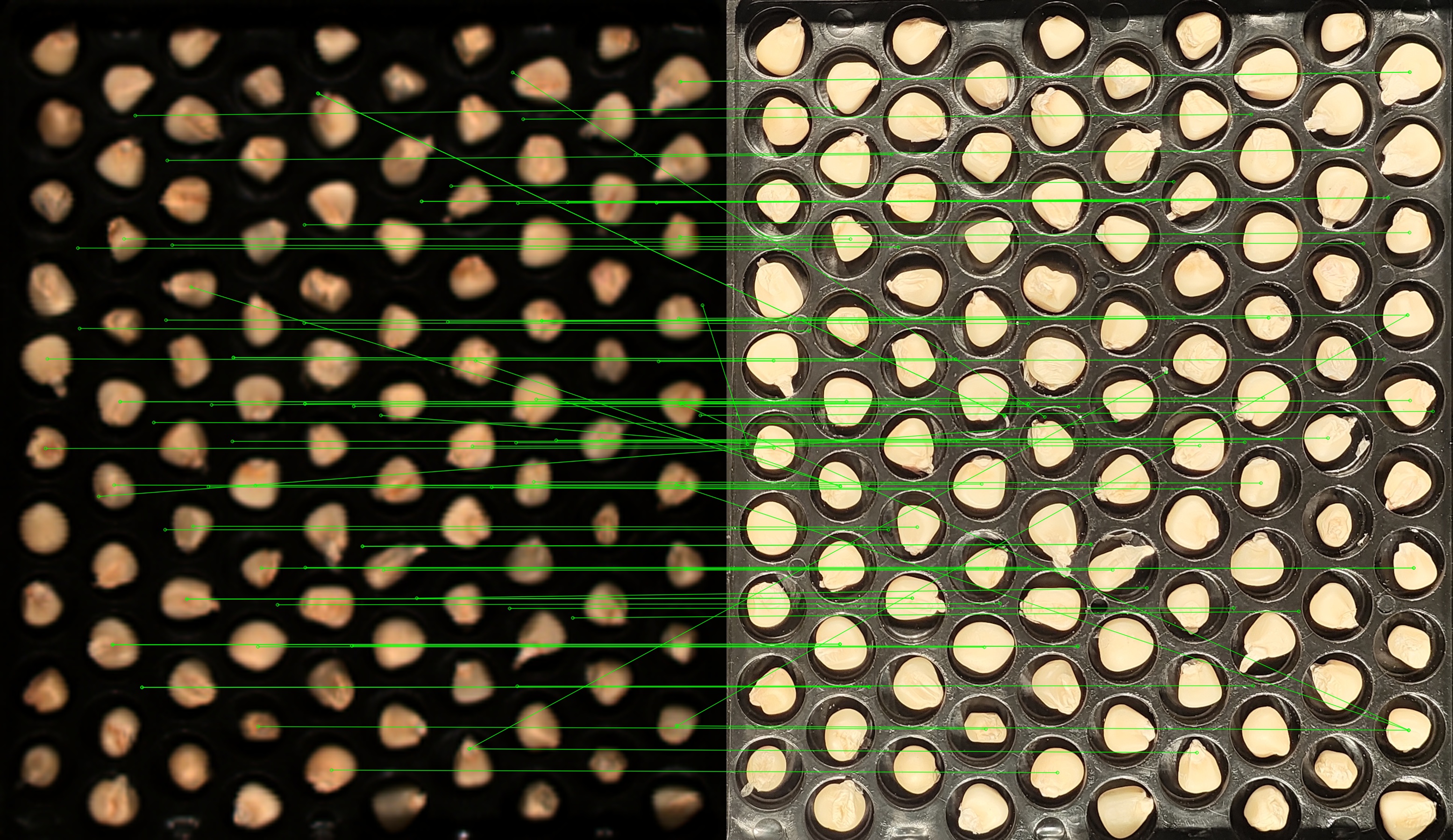

Supplement: Supplementary file 1 [file mmc1.zip › Spatial Registration/Zhuyunuo No.1-1/Zhuyunuo No.1_2_matches_viz.jpg]

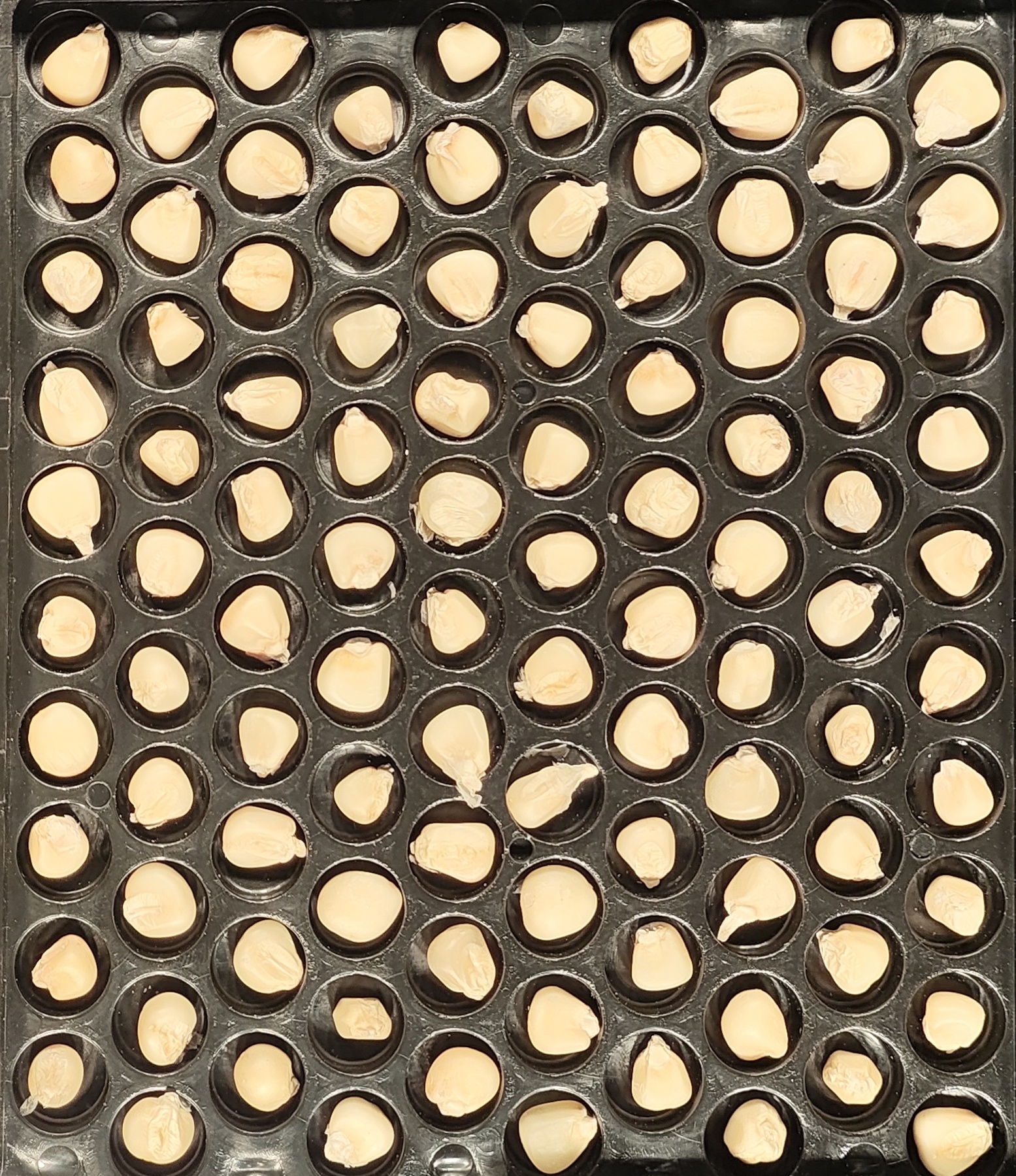

Supplement: Supplementary file 1 [file mmc1.zip › Spatial Registration/Zhuyunuo No.1-1/Zhuyunuo No.1_2_rgb_reg_high.jpg]

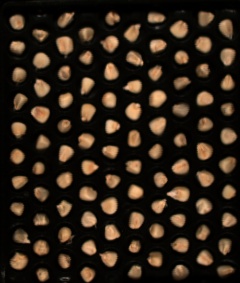

Supplement: Supplementary file 1 [file mmc1.zip › Spatial Registration/Zhuyunuo No.1-2/Zhuyunuo No.1_3_hsi_pseudo.jpg]

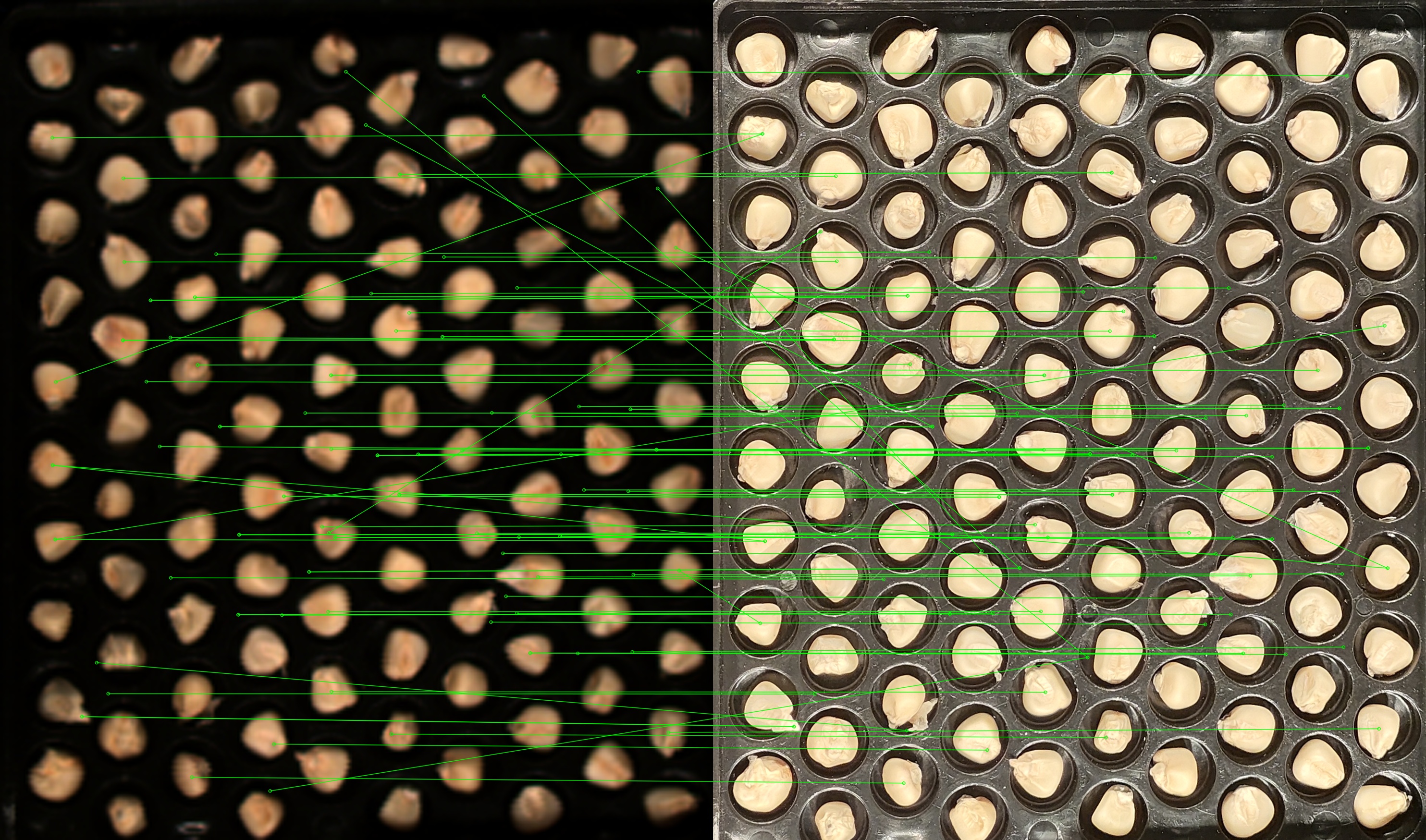

Supplement: Supplementary file 1 [file mmc1.zip › Spatial Registration/Zhuyunuo No.1-2/Zhuyunuo No.1_3_matches_viz.jpg]

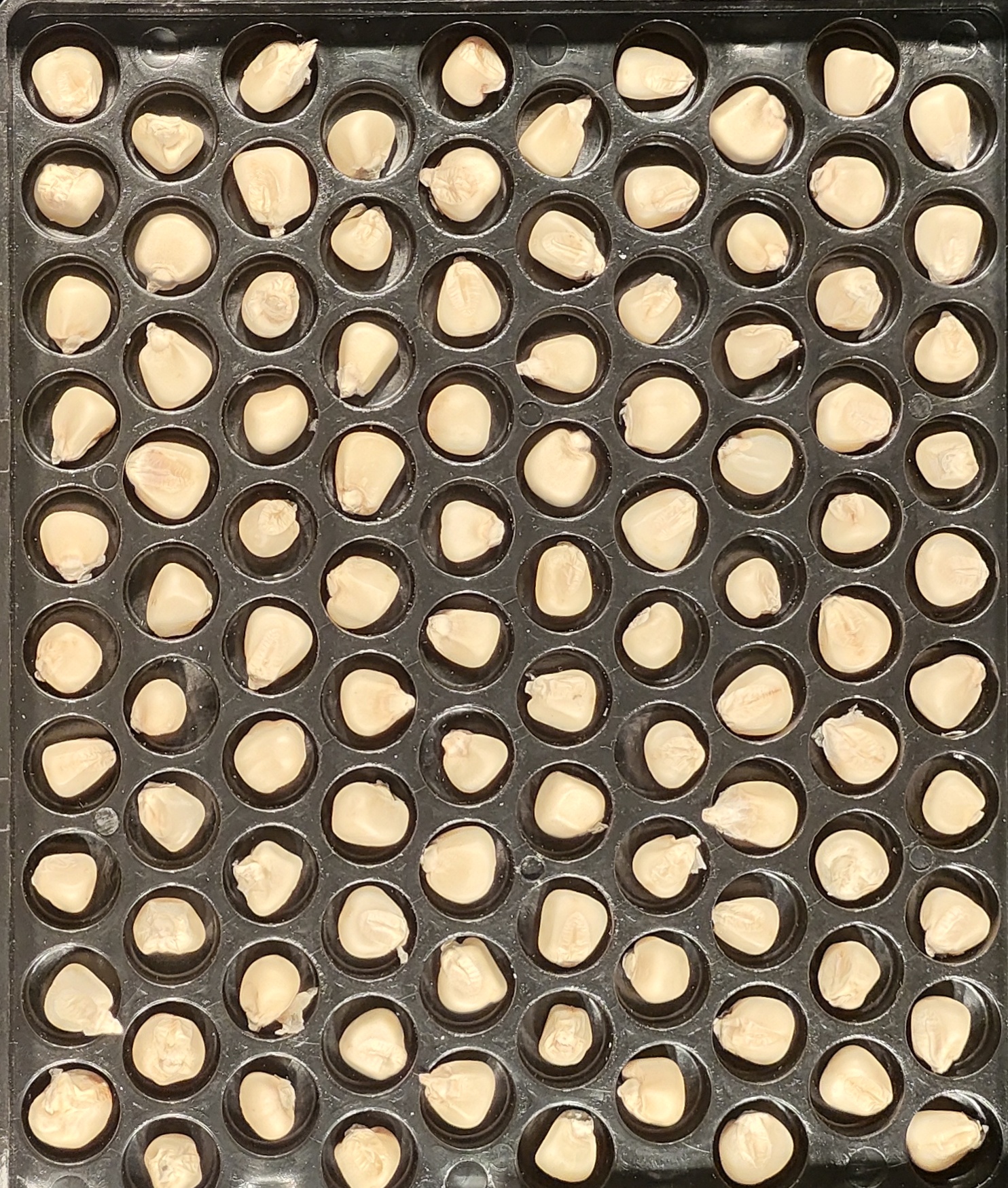

Supplement: Supplementary file 1 [file mmc1.zip › Spatial Registration/Zhuyunuo No.1-2/Zhuyunuo No.1_3_rgb_reg_high.jpg]

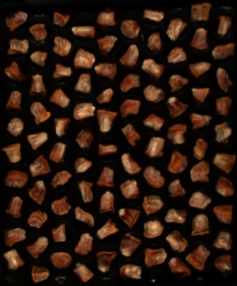

Supplement: Supplementary file 1 [file mmc1.zip › Spatial Registration/Zhuyutian No.1-1/Zhuyutian No.1_hsi_pseudo.jpg]

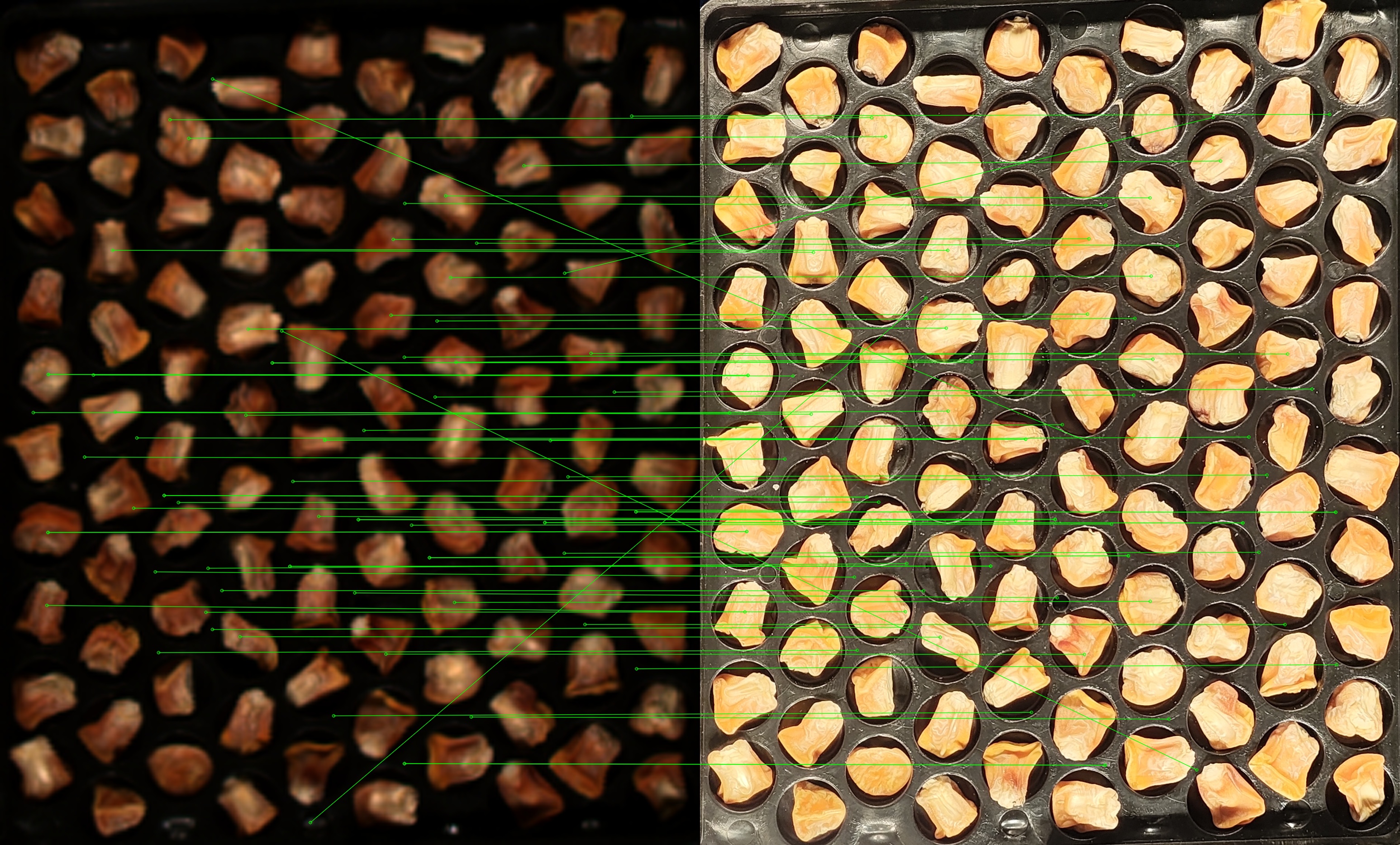

Supplement: Supplementary file 1 [file mmc1.zip › Spatial Registration/Zhuyutian No.1-1/Zhuyutian No.1_matches_viz.jpg]

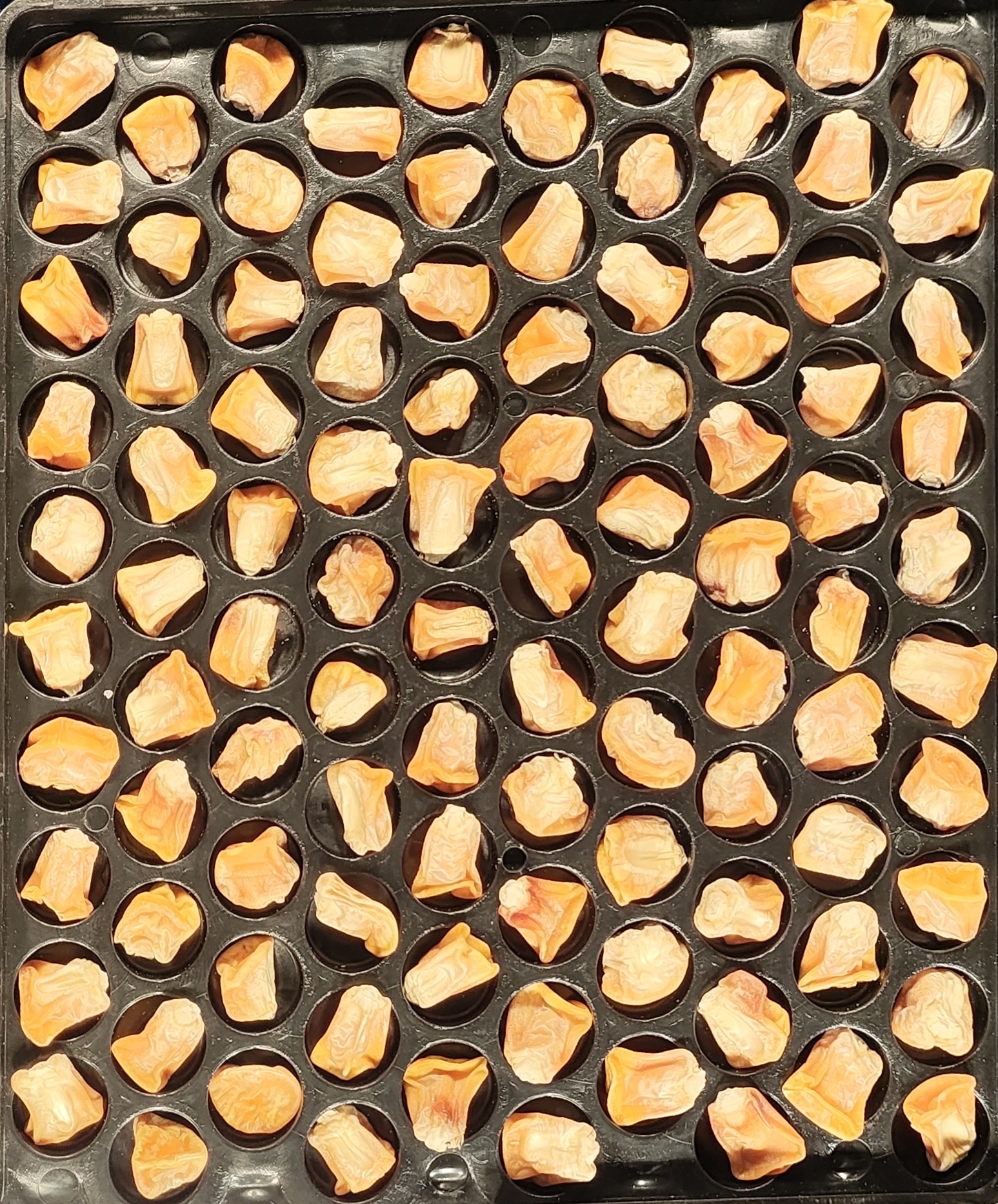

Supplement: Supplementary file 1 [file mmc1.zip › Spatial Registration/Zhuyutian No.1-1/Zhuyutian No.1_rgb_reg_high.jpg]

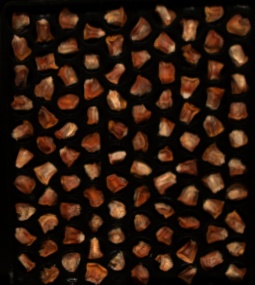

Supplement: Supplementary file 1 [file mmc1.zip › Spatial Registration/Zhuyutian No.1-2/Zhuyutian No.1_3_hsi_pseudo.jpg]

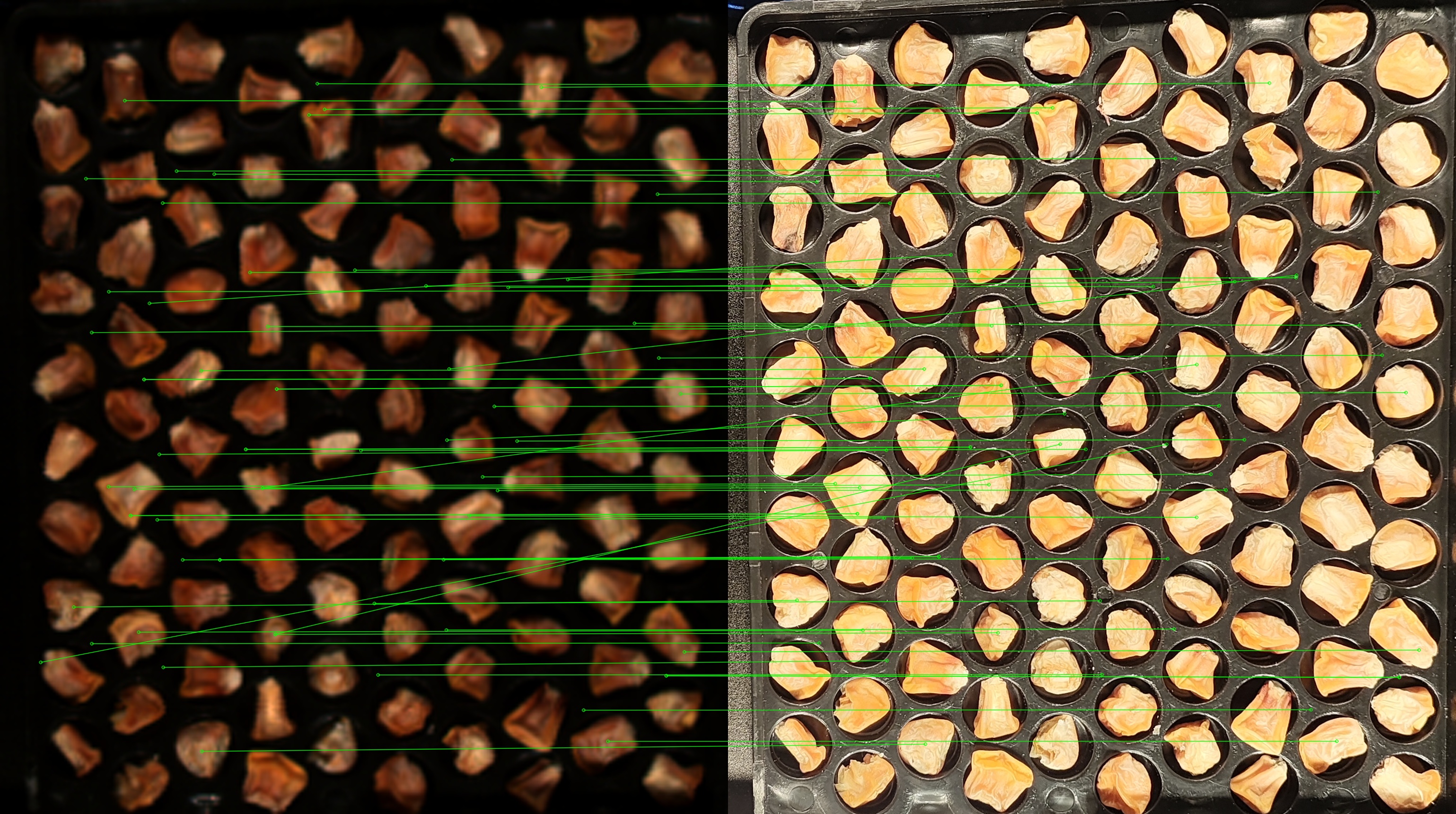

Supplement: Supplementary file 1 [file mmc1.zip › Spatial Registration/Zhuyutian No.1-2/Zhuyutian No.1_3_matches_viz.jpg]

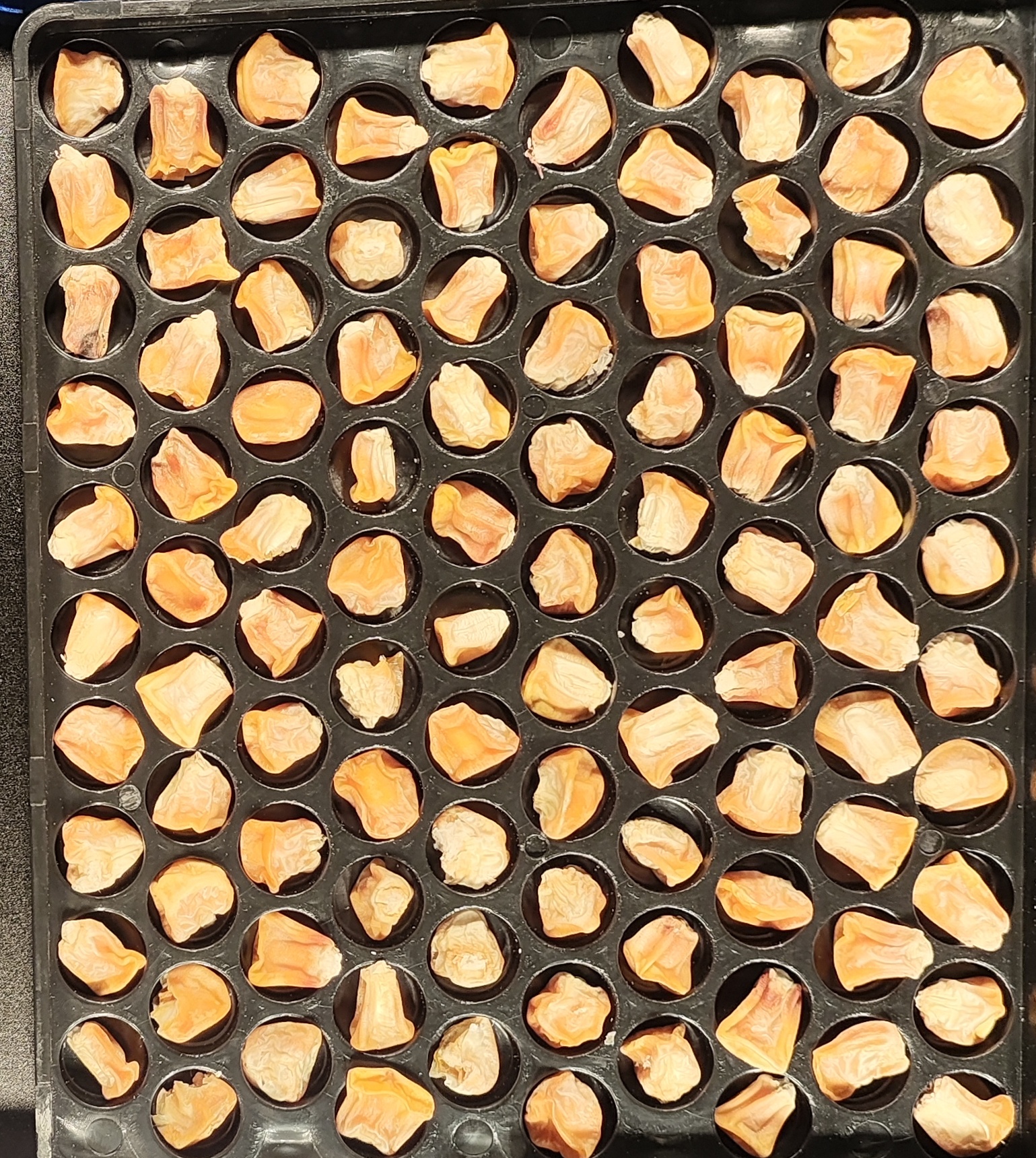

Supplement: Supplementary file 1 [file mmc1.zip › Spatial Registration/Zhuyutian No.1-2/Zhuyutian No.1_3_rgb_reg_high.jpg]
